# Structural basis of seamless excision and specific targeting by *piggyBac* transposase

**DOI:** 10.1038/s41467-020-17128-1

**Published:** 2020-07-10

**Authors:** Qiujia Chen, Wentian Luo, Ruth Ann Veach, Alison B. Hickman, Matthew H. Wilson, Fred Dyda

**Affiliations:** 1 Laboratory of Molecular Biology, National Institute of Diabetes and Digestive and Kidney Diseases, National Institutes of Health, Bethesda, MD 20892 USA; 2 Department of Medicine, Division and Nephrology and Hypertension, Vanderbilt University Medical Center, Nashville, TN USA; 3 Department of Veterans Affairs, Nashville, TN USA; 4 Department of Pharmacology, Vanderbilt University, Nashville, TN USA

**Keywords:** Transposition, Cryoelectron microscopy

## Abstract

The *piggyBac* DNA transposon is used widely in genome engineering applications. Unlike other transposons, its excision site can be precisely repaired without leaving footprints and it integrates specifically at TTAA tetranucleotides. We present cryo-EM structures of *piggyBac* transpososomes: a synaptic complex with hairpin DNA intermediates and a strand transfer complex capturing the integration step. The results show that the excised TTAA hairpin intermediate and the TTAA target adopt essentially identical conformations, providing a mechanistic link connecting the two unique properties of *piggyBac*. The transposase forms an asymmetric dimer in which the two central domains synapse the ends while two C-terminal domains form a separate dimer that contacts only one transposon end. In the strand transfer structure, target DNA is severely bent and the TTAA target is unpaired. In-cell data suggest that asymmetry promotes synaptic complex formation, and modifying ends with additional transposase binding sites stimulates activity.

## Abstract (web summary)

*PiggyBac* is a transposon used in genome engineering that does not leave excision footprints. Here the authors determine the structures of two complexes in which the *piggyBac* transposase is bound to DNA representing different steps of the transposition reaction, providing a basis for how the transposition reaction proceeds.

## Introduction

Transposons are mobile genetic elements that can move from one position to another in the genome or between host genome and foreign DNA^1^. They make up a large percentage of most eukaryotic genomes^1–3^, where they have played important roles in genome evolution and the establishment of novel cellular functions and pathways^4^. Although their movement has been severely constrained in humans, largely through inactivation, transposition has been linked to the development of specific diseases^5,6^.

**Fig. 1:**
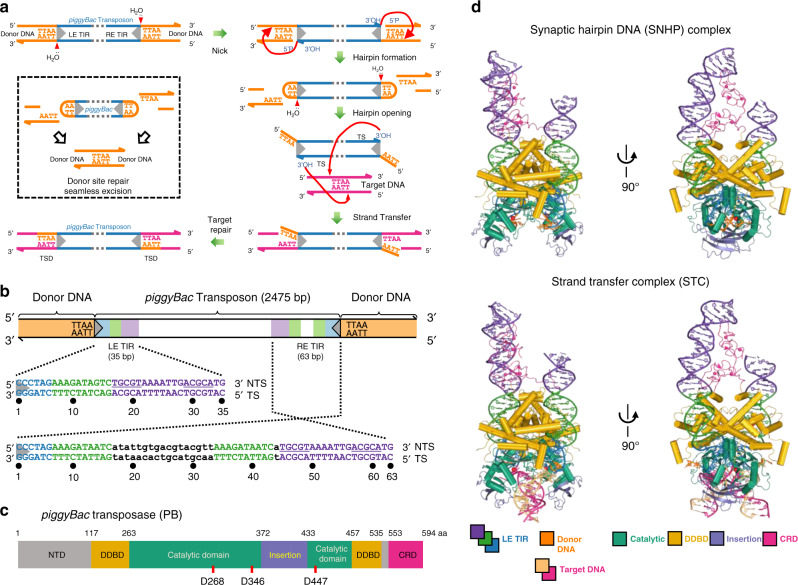
Overview of *piggyBac* transposition. **a** Mechanism of *piggyBac* (*pB*) transposition. Hydrolysis by the *piggyBac* transposase (PB) liberates the 3′-OH on the DNA strand that will be integrated. This is followed by transesterification in which this 3′-OH attacks flanking DNA four nt from the transposon end forming a DNA hairpin. For subsequent integration, PB opens the hairpin at each end in another hydrolytic step, leaving a four nt TTAA overhang; this is followed by a second transesterification step that joins each end to target DNA. At the empty donor site (inset), complementary DNA strands allow for seamless repair. LE left end, RE right end, TIR terminal inverted repeat, TSD target site duplication. **b** Schematic of *pB* transposon flanked by TTAA, and sequence and organization of the LE and RE TIRs. NTS non-transferred strand, TS transferred strand. **c** Domain organization of PB. The catalytic domain contains the conserved DDD motif (in red). NTD N-terminal domain. Dimerization and DNA-binding domain (DDBD). CRD C-terminal cysteine-rich domain. Gray indicates disordered regions in the structure. **d** Overall structures of the synaptic hairpin DNA (SNHP) complex and strand transfer complex (STC).

DNA transposons, most notably *Sleeping Beauty* (*SB*) and *piggyBac* (*pB*), have been intensively exploited as tools for genome engineering and therapeutic applications^7–10^. Among eukaryotic DNA transposons, only *pB* is known to specifically integrate at TTAA sites and to exhibit the property of seamless excision whereby the genomic gap produced when the transposon is excised can be repaired precisely, without need for any DNA synthesis (Fig. 1a)^11,12^. *pB* has proved to be extremely versatile and the lack of a DNA footprint left behind after its transposition is a unique and valuable property^13–16^. It is used in non-viral vectors for transgenesis^17,18^, gene therapy^7,19^, insertional mutagenesis^20^, and genetic screens^21–23^. It has also found application in novel therapeutic strategies including CAR T-cell engineering^24–26^, CRISPR/Cas-mediated gene therapy^27–29^, and human induced pluripotent stem cells (iPSC) engineering^30–32^. Useful variants have been developed through random mutation including a hyperactive *pB* transposase called hyPBase^33^ as well as one that can excise *pB* but cannot integrate it^34^.

Although *pB* (Fig. 1b) was isolated from the cabbage looper moth *Trichoplusia ni* over 30 years ago^11^, no structural information is available that would explain its excision and targeting properties or provide a rational basis to understand how mutations affect its activities. *pB* contains a single open-reading frame (ORF) encoding its 594 amino-acid transposase (PB) (Fig. 1c) flanked by terminal inverted repeats (TIRs) (Fig. 1b). The *pB* TIRs are asymmetric; sufficient for certain in vitro and in vivo activities are the 35 bp left end TIR (LE TIR or LE35) and 63 bp right end TIR (RE TIR or RE63)^35^. Although LE35 and RE63 have short sequences and repeats in common (shown in blue, green, and purple in Fig. 1b), the relative organization of these differs and RE63 has a 17 bp insertion and a sequence duplication between segments relative to LE35 (Fig. 1b, in white and green, respectively), a puzzling arrangement.

PB is a cut-and-paste DNA transposase, proposed to be a member of the RNaseH-like or retroviral integrase superfamily^36^. PB-catalyzed transposition proceeds through a series of hydrolysis and transesterification steps^35^ that generate an excised intermediate in which DNA hairpins protect the transposon ends (Fig. 1a). The reaction sequence differs from those of other eukaryotic DNA transposases, such as members of the *Tc1/mariner* and *hAT* superfamilies, or the *Transib* precursor to RAG1 of the V(D)J recombination system^37–40^, but is identical to the prokaryotic IS*4* family of insertion sequences and transposons such as Tn*5*^41^. Despite this shared reaction pathway, however, only *pB* precisely targets TTAA sequences^36^.

Motivated by *pB*’s unique properties and its importance in established and emerging applications, we have determined two ab initio single-particle cryo-electron microscopy (cryo-EM) structures of PB in complex with DNA: a synaptic complex that contains the hairpinned DNA intermediates (synaptic hairpin complex (SNHP)) and a strand transfer complex (STC) in which two TIRs have been integrated into target DNA. The structures explain the basis of seamless excision and targeting, and together with biochemical and in-cell transposition data, suggest a transposition model that requires asymmetric TIRs in order to form the active synaptic complex in vivo.

## Results

### Mammalian-expressed PB is active in vitro

PB expressed and purified from mammalian cells forms dimers as judged by SEC-MALS (Supplementary Fig. 1a)^42^. In vitro, as previously demonstrated for PB expressed in *E. coli*^35^, it catalyzed hairpin formation and resolution with oligonucleotide TIR substrates (Supplementary Fig. 1b). It also catalyzed double-ended integration of pre-cleaved transposon ends into a supercoiled (SC) pUC19 target plasmid (Supplementary Fig. 1c). For example, incubation with a pre-cleaved 35 bp LE TIR oligonucleotide (LE35) and pUC19 generated products consistent with single-end (SE) and double-end (DE) integration (Supplementary Fig. 1c). Under assay conditions with a protein:TIR ratio of 1:2, plasmid integration was less efficient when a pre-cleaved 63 bp RE TIR (RE63) was used, or when LE35 and RE63 were mixed at equal molar ratio (Supplementary Fig. 1c).

Present in both LE35 and RE63 is a palindromic-like 19-bp internal repeat (the purple box sequence in Fig. 1b) that is the binding site for the C-terminal cysteine-rich domain (CRD; residues 553–594) of PB^43^. The CRD is required for PB activity and has been proposed to be the driver of TIR binding. When we probed the effect of sequentially truncating RE63 to remove the CRD-binding site (Supplementary Fig. 1d), we were surprised to observe increasing stimulation of integration into either linearized or SC pUC19 as the RE was shortened to RE24. Further deletion to RE16 largely abolished integration.

To confirm that mammalian-expressed PB integrated with its expected target site specificity, we generated a linear mini-transposon in which LE35 and RE63 TIRs flanked a Kan resistance gene, and used this as a substrate for in vitro integration into SC pUC19. The reaction products were purified and transformed into *E. coli*, allowing us to select for Amp+Kan+ colonies corresponding to integrated mini-transposons. Sequencing confirmed that only TTAA tetranucleotides had been targeted: from 10 sequenced colonies, we detected four mini-transposon integration events corresponding to insertion at TTAA sequences at bp 635–638, bp 1568–1571, bp 1582–1585, and bp 2646–2649 of pUC19.

### Cryo-EM structure determination

For structural studies, we investigated the biophysical properties of a variety of PB/TIR complexes. In most cases, analytical size-exclusion chromatography (SEC) revealed multidisperse elution profiles with overlapping peaks. However, when PB was complexed with LE35 hairpin intermediates (Supplementary Fig. 2a) in the presence of Ca^2+^ (which does not catalyze hairpin opening, Supplementary Fig. 1b), it was possible to isolate a monodisperse population containing SNHP (Supplementary Fig. 2b). We also assembled a STC that contained PB, Ca^2+^, and 37 bp LE TIRs covalently joined to target DNA (Supplementary Fig. 3). As DNase I footprinting data suggested that PB interacts with 4–5 bp of flanking DNA beyond its TTAA target^43^, target DNA included 11 bp on either side of the central TTAA (Supplementary Fig. 3a). To avoid base-pairing during substrate annealing between the target sequence and the four nt TTAA overhang derived from hairpin opening (Fig. 1a), we substituted the overhang with the mutated sequence CCGG (Supplementary Fig. 3a). It has been shown that the four nt overhang is not required for TTAA target site selection in vitro^35^ nor do mutated flanking sequences affect specific integration into TTAA sites in vivo^44^. Our attempts to prepare stable monodisperse complexes containing one LE TIR and one RE TIR have not yet been successful.

We collected single-particle cryo-EM data on both complexes. For each, particle projections showed a wide distribution of different orientations in the micrographs and a number of distinct two-dimensional (2D) classes (Supplementary Fig. 4a, b). For the SNHP complex, we applied a mask around the CRD for masked three-dimensional (3D) classification which improved the resolution of the reconstructed density map to 3.66 Å (Supplementary Fig. 4a, c). For the STC, we used two rounds of 3D classification and one round of masked 3D classification, resulting in a 3.47 Å density map (Supplementary Fig. 4b, e) with better map quality when compared with the SNHP complex, most likely due to the additional stabilizing effect of the target DNA. While parts of the maps were clearly two-fold symmetric, the maps as a whole had no rotational symmetry (Supplementary Fig. 4a, b). Therefore, all processing was done without applying symmetry.

An NMR structure has been determined for the CRD (PDB 5LME)^43^, but as there was no known homologous structure available for the rest of PB, ab initio atomic models were built into Phenix-sharpened^45^ potential density maps. For the SNHP complex, we used Phenix’s map to model module^46^ and fragments were connected manually. The NMR model of the CRD dimer was consistent with the potential density in the region and was used as a starting model. We used density-guided rebuilding tools of Rosetta^47^ to complete and verify the trace and register of the model that was subsequently refined using Rosetta and Phenix. The STC model was built based on the SNHP model. Representative regions of the potential density maps and model fits are shown in Supplementary Figs. 5 and 6. The final models contain residues 117–594, consistent with the prediction by the PONDR server^48^ that the N-terminal 110 residues are largely intrinsically disordered.

### Overall structures of PB transposase complexes

**Fig. 2:**
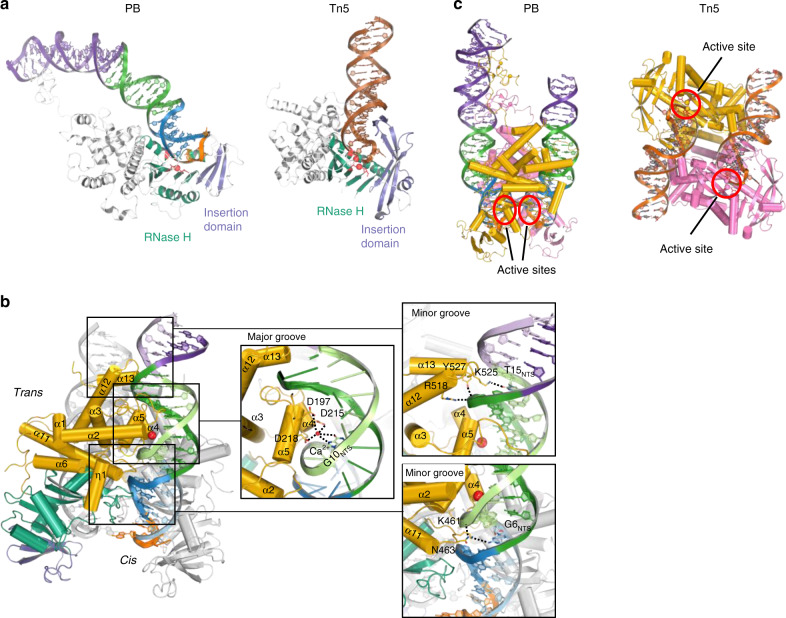
Comparison of PB and Tn*5* and details of PB TIR recognition. **a** Comparison of domain organization in PB SNHP and Tn*5* transpososomes. RNaseH-like catalytic domains are in green, with active site residues highlighted in red. Insertion domains are colored in violet. The insertion domain of PB contains fewer β-strands than that of Tn*5*. **b** The DDBD interacts with LE TIR *in* *trans*. The α-helices comprising DDBD widen the major groove of the repeat shown in green. The red sphere is a Ca2+ ion in the DDBD/DNA interface. Other parts are colored as in Fig. 1d. **c** Comparison of TIR binding by PB (TIR DNAs are parallel; shown for the SNHP complex) and Tn*5* transposase (anti-parallel TIRs; PDB 1MUS). Both transposases demonstrate *cis* and *trans* protein/DNA interactions. *pB* DNA colors are as in Fig. 1d. Tn*5* TIR DNA is brown. Individual monomers are shown in yellow and pink. Active site locations are indicated (red).

In both the SNHP and STC complexes, an asymmetric PB dimer synapses two approximately parallel TIRs (Fig. 1d). As predicted^35^, the catalytic domain (residues 263–457, separated by an insertion domain from residues 372 to 433) possesses the fold of the RNaseH-like superfamily of transposases. After the fifth β-strand of the catalytic domain, a three β-stranded insertion domain interrupts the RNaseH-like fold (Fig. 2a and Supplementary Fig. 7), the same topological location as in all other DDE/D transposases featuring insertion domains^49^. The convergence of residues 117–263 and 457–535 forms a structurally unique^50^ all-α-helical domain (denoted Dimerization and DNA-binding domain, DDBD) knitting the protein together and which interacts with TIR bp 7–16 (Fig. 2b). The C-terminal end of the DDBD is connected to the CRD through an extended linker that exhibits weak density.

In both complexes, the DDBD, catalytic and insertion domains collaborate to synapse two LE TIRs and direct the scissile phosphates to the active sites comprised of D268, D346, and D447 where a Ca^2+^ ion is bound by the carboxylates of D268 and D346 (Supplementary Fig. 6a, b). A bridging oxygen from the scissile phosphate also forms part of the Ca^2+^ ligand sphere, suggesting that PB follows the paradigm of the two metal-ion-dependent chemical mechanism^51,52^.

In the SNHP complex, the horseshoe-shaped dimer formed by the DDBD, catalytic and insertion domains creates a wide channel whose sides are lined with β-hairpin loops largely contributed by the insertion domain (Fig. 1d). In the STC, this channel is filled with the target DNA (Fig. 1d). The SNHP and STC complexes are very similar and can be superimposed at 1.22 Å rmsd over 933 α-carbon positions. Upon target DNA binding, the channel formed by the catalytic and insertion domains narrows by about 3 Å as the two halves of the transpososome close down upon it, although we cannot rule out the possibility that this observation was influenced by the selection of the 3D classes.

### CRD binding generates asymmetry and bends one TIR

**Fig. 3:**
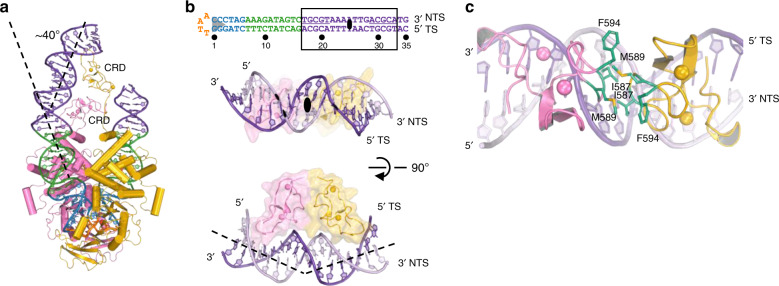
C-terminal CRD binding generates asymmetry. **a** Cysteine-rich domain (CRD) dimer binds the palindromic 19-bp internal repeat of only one LE TIR, resulting in a ~40° kink of the DNA. DNA is colored as in Fig. 1d. Individual monomers are shown in yellow and pink. Bound Zn^2+^ ions are rendered as spheres. **b** Close-up view of the CRD dimer–DNA interaction. Top, sequence and numbering of hairpin DNA in the SNHP complex. The palindrome within bp 17–33 (purple) is underlined, and the two-fold symmetry axis is indicated. Bottom, orthogonal views of the CRD dimer. DNA binding by the CRDs narrows the minor groove centered at ~bp 25. NTS non-transferred strand, TS transferred strand. **c** Hydrophobic residues at the CRD dimer interface.

The DDBDs, catalytic and insertion domains, and the first 16 bp of the LE TIRs bound by them obey two-fold symmetry (Fig. 1d and Supplementary Fig. 4) (extending also to the target DNA in the STC), yet overall the complexes are markedly asymmetric as the two CRDs form a dimer (Fig. 3) that binds bp 17–33 of only one of the LE TIRs (corresponding to the 19-bp palindromic internal repeat, in purple in Fig. 1b). There is no detectable density around the equivalent palindromic internal repeat sequence of the other LE TIR and, despite the weaker DNA density (presumably due to the lack of the stabilizing effect of bound CRDs), it is clear that it is not bent (Supplementary Fig. 4). The CRD monomers refined against the cryo-EM maps are consistent with the cross-brace Zn finger structure determined by NMR^43^, although not identical due to side chain rearrangements at the hydrophobic CRD dimer interface (Fig. 3c).

CRD binding causes a ~40° bend in the TIR due to the insertion of the CRDs into two adjacent widened major grooves, accompanied by a significant narrowing of the intervening minor groove centered around bp 25 (Fig. 3a, b). The purple box of LE TIR DNA contains a canonical A-tract (bp 22-25) that appears to display propeller twist and has a narrowed minor groove, features previously noted for A-tracts^53^. The CRD dimer is approximately two-fold symmetric with a rotation axis perpendicular to the DNA and centered at the minor groove in the middle of the CRD-binding site (Fig. 3b). At the level of the CRD monomer, the observed mode of DNA binding is broadly consistent with the model derived from NMR including the identification of Y558, R567, and K569 as crucial residues for specific recognition^43^, but the NMR model did not predict the severe DNA bend induced by CRD dimerization.

### TIR recognition close to the transposon end

Although the DDBDs interact with both TIRs, most of the protein/DNA interactions are *in trans*, such that the interactions of one monomer with a TIR direct its end into the active site of the other monomer (Fig. 2b). Such *trans* arrangements are common in transpososome assemblies^40,54–56^. The parallel TIR configuration is in sharp contrast with the arrangement within the Tn*5* transpososome^54^ (Fig. 2c) that catalyzes the same reaction sequence. Remarkably, the two transpososomes are organized in completely different ways, reflecting two very different modes of transposase dimerization, with the Tn*5* TIRs almost antiparallel and essentially straight (Fig. 2c). However, the two transposases do share the feature of an entirely β-stranded insertion domain (Supplementary Fig. 7). While the DD(E/D) catalytic and parts of the insertion domains of PB and the Tn*5* transposase can be aligned (1.99 Å rmsd over 182 α-carbons), the sequence identity in this region is only 9%.

In both PB complexes, the catalytic domain interacts with the minor groove closest to the transposon end (bp 2–6) through a rich set of interactions, including two α-helices linked by G444 and G445 (α9–α10 in Supplementary Fig. 7) that turn and follow the curve of the minor groove. The α10 helix carries D447 that together with D268 and D346 form the catalytic triad (Supplementary Fig. 6a, b). One turn away from the tip of the transposon, the DDBD binds the TIR *in trans*, widening the major groove (bp 9–14) through the insertion of three different parts of the protein (i.e., α4, α5, and a loop between α2 and α3) (Fig. 2b). In addition, the two adjacent, narrowed minor grooves (bp 5–8 and bp 15–18) are also recognized by elements of the DDBD (Fig. 2b). There is also a curious arrangement in which three carboxylates (D197, D215, and D218) coordinate a divalent metal ion (Ca^2+^) that is also coordinated by several bases in the widened major groove (Fig. 2b).

**Fig. 4:**
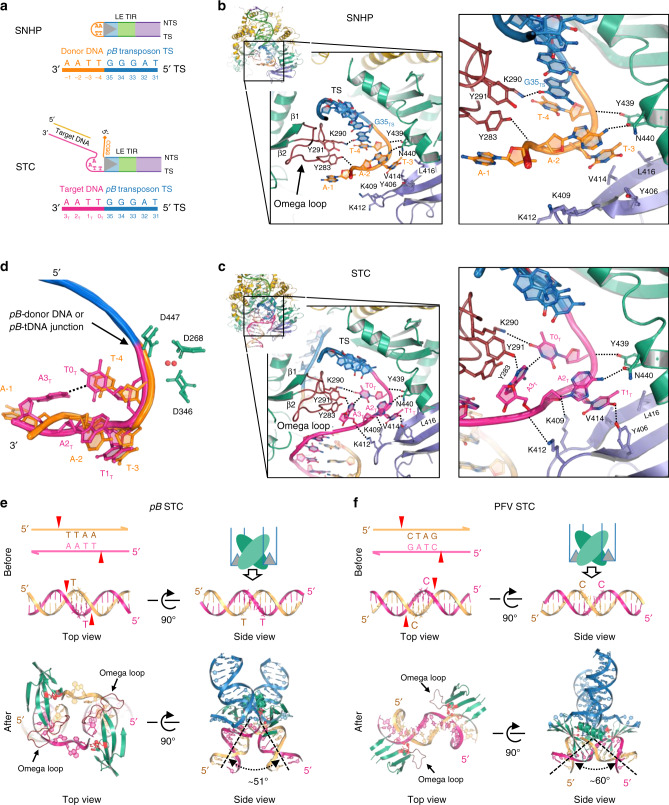
SNHP and STC of PB reveal the mechanistic link between seamless excision and TTAA targeting. **a** Numbering system used for SNHP and STC DNA substrates. **b**, **c** Detailed protein–DNA interactions of TTAA tetranucleotides in the SNHP and STC. The interactions involve the omega loop (brown), the catalytic domain (green), and the insertion domain (purple). In STC, A3_T_ (equivalent to the flipped base A-1 in SNHP) is hydrogen bonded to T0_T_, shown as a dashed line. For clarity, the NTS is not shown. Protein domains and DNA are colored as in Fig. 1d. **d** Superposition of the TTAA tetranucleotides in SNHP and STC. Except A-1 in SNHP and A3_T_ in STC, the other bases have similar conformations in both complexes. In the SNHP, A-1 is flipped out whereas, in the STC, A3_T_ is hydrogen-bonded to T0_T_, represented as a black dashed line. **e**, **f** Comparison of target DNA integration of PB and PFV. PB binds and integrates into target DNA through the minor groove. Top, red triangles indicate staggered sites for integration (labeled as nucleotide T for PB and C for PFV) and the relative positions of the catalytic domains. The side view cartoons show how a PB dimer approaches target DNA towards the minor groove while PFV integrates through the major groove. Bottom, structure of the catalytic domains (green) interacting with target DNA. Due to severe distortion of the DNA backbone, the PB target site bases are unpaired, with two bases flipped out. Unlike in PB STC, the 4-bp PFV target site remains base-paired. The omega loops in PFV has no interactions with the target DNA. PFV prototype foamy virus (PDB ID: 4E7L).

Compared to the PB transposase, that of Tn*5* has additional β-strands, and β7 and β8 of Tn*5* form a β-hairpin that lies deep in the major groove just adjacent to the DNA hairpin (Fig. 2a and Supplementary Fig. 7). For Tn*5*, mutations in this β-hairpin affect all steps of transposition^57^. The same role in PB is served by an omega loop between the first and the second β-strands of the catalytic domain that interacts with the hairpin and hairpin-proximal major groove through R275, Y283, and K290 *in trans* (Fig. 4b).

### SNHP complex and DNA hairpin recognition

**Fig. 5:**
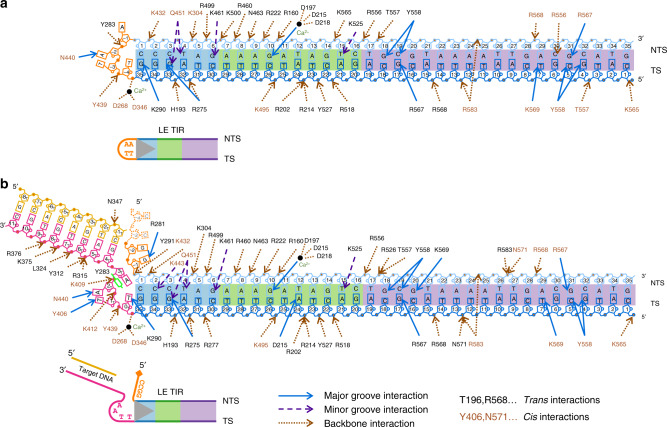
Schematic diagram of protein–DNA interactions. Numbers in pentagons represent the positions of the nucleotides. Letters in the boxes are types of nucleobases. In the LE TIR hairpin DNA, the nucleotides of the TIR portion (NTS and TS) are from 1 to 35 (5′–3′). The hairpin TTAA (orange) is numbered −4 to −1 (5′–3′). In the strand transfer complex DNA, the TIR portion is numbered the same as in the LE TIR hairpin DNA. Upon integration, the TS strand is covalently joined to the target DNA started from position 0 of the target DNA (0_T_); thus, the bottom strand of the target DNA (pink) is from 0_T_ to 11_T_ (5′–3′). The top strand of target DNA (yellow) is indicated as −8_T_ to −1_T_ (5′–3′). The flap donor DNA (orange, CCGG) is numbered −4 to −1 (5′–3′), consistent with the hairpin DNA scheme.

In the SNHP complex, all the nts of the transposon end are base-paired whereas none of the four nts of the hairpin loop (numbered as A-1, A-2, T-3, T-4), derived from the flanking TSD DNA, are base-paired (Supplementary Fig. 5c). Multiple elements contribute to the stabilization of the hairpin. Backbone phosphates of the hairpin loop interact with Y439 from the catalytic domain *in cis*, and Y283 from the omega loop *in trans* (Figs. 4b and 5). There is also a base-specific interaction between N440 and A-2 *in cis* (Figs. 4b and 5). In addition, the methyl group of T-3 is in a hydrophobic pocket formed by V414/L416/Y439 of the insertion domain *in cis*.

Although A-1 is completely flipped out, there do not appear to be aromatic stacking interactions with PB side chains. This is in contrast to the hairpin loop of Tn*5* in which the collaboration of two tryptophan residues is needed to form the tight hairpin on each transposon end, one pushing out a thymine base from the non-transferred strand (NTS) and preventing its return to the helix and another stacking against it once it is flipped out^54,58,59^. This difference is likely because in Tn*5*, the tightest possible hairpin is formed by the attack of the transferred strand (TS) terminal 3′-OH on the terminal nt of the NTS directly opposite. In *pB*, the TS terminal 3′-OH instead attacks flanking DNA, four nt from the transposon end of the opposite strand (Fig. 1a), and the structure suggests that this is long enough that a hairpin can readily form. The active site area of PB is more open when compared to that of Tn*5*, consistent with the ability to accommodate a longer hairpin loop and to allow the conformation change of the NTS that might be required to bring the scissile phosphate to the active site. The 4 nt long hairpin loop is stabilized by a set of interactions with the omega loop *in trans*, which are absent in Tn*5*.

### STC complex reveals target DNA recognition and integration

In the STC, all nts of the transposon DNA are base-paired as are the target nts except for the bps that comprise the specific TTAA tetranucleotide (numbered as T0_T_, T1_T_, A2_T_, A3_T_), all four of which are unpaired (Supplementary Figs. 5d and 6c). It appears that specificity for the TTAA target sequence arises from its conformation as an ssDNA segment. The two ssDNA segments are stabilized by an elaborate network of protein/DNA and DNA/DNA interactions (Figs. 4c and 5) organized by the omega loop. Remarkably, relative to the hairpin loop seen in the SNHP complex, many of the same protein residues are involved and the conformations of T0_T_, T1_T_, and A2_T_ are essentially superimposable on T-4, T-3, and A-2 of the hairpin DNA, respectively (Fig. 4d). The major change is with A3_T_ (equivalent to A-1 in the hairpin loop) which is not flipped out but, rather, turned back towards the other target bases and H-bonded through its N1to N3 of T0_T_ (Figs. 4c, d and 5) and stacked against Y283. To allow the reaction to proceed from the hairpin-bound state to one poised to capture target, it appears that hairpin resolution is followed by the movement of the resulting flap out of the active site. Then, a drastically distorted and unpaired target TTAA tetranucleotide can be bound. As the structures reveal, the key to *pB* transposition is that the backbones of the TTAA tetranucleotide in both the hairpin and target DNA adopt a very similar conformation (Fig. 4d). The role of the PB protein is therefore to enforce this conformation at both steps of the reaction.

The density is poor for the tetranucleotide flap CCGG after the first two nucleotides, presumably due to flexibility. Minimal interactions with the flap are consistent with observations that the flap is not necessary for PB reactions in vitro^35^ (Supplementary Fig. 1c, d), and that the exact 4 bp sequence of flanking DNA (which becomes the flap) is not important in vivo^35,44^. We observe interactions between several amino acid residues and backbone phosphates for five bp beyond the target TTAA, and the side chains of R372 and R376 are inserted into the minor groove of the flanking region (A5_T_–G8_T_, Fig. 5b).

The STC structure suggests that PB achieves target specificity by stabilizing the strand transfer product through the ssDNA form of TTAA. This unusual mode of transposon target selection is in line with the lack of a region of PB recognizable in the structure as a potential dsDNA TTAA target recognition domain. Although the role of the disordered N-terminal region remains to be established, the predicted isoelectric point of residues 1–117 is 4.7, making it highly unlikely that this region contributes to DNA binding.

PB integration occurs symmetrically, and the configuration of the dsDNA regions of the target that flank TTAA suggests that it must be severely bent before integration (Fig. 4e). Bent target DNA is a common feature of most DNA transpososome and intasome structures^40,49,56,60–64^. Strikingly, a unique aspect of the PB STC is that integration occurs at staggered target DNA phosphates across the minor groove (Fig. 4e). In all other known transpososome structures, integration has invariably been observed to occur across the major groove, as for example in the PFV intasome that also integrates with a four bp TSD (Fig. 4f, PDB 4E7L^60^). In PFV, the strand transfer sites (C, Fig. 4f, top) are easily accessed by bending the target DNA to widen the major groove to fulfill the distance between active sites. In the PB STC, however, the strand transfer sites (T, Fig. 4e, top) are opposite the bound PB dimer. Simply bending the target DNA is not sufficient to allow the target scissile phosphodiester bonds to reach the active sites that are located deep within the catalytic domains. Instead, to fit the orientation of the active site DDD motif, coordinated metals, and the 3′-OH of the TS during the strand transfer reaction, TTAA tetranucleotides are melted and drastically distorted (Fig. 4e). Interestingly, the omega loop of PB interacts with target DNA, facilitating the melting of TTAA (Fig. 4e, bottom). The equivalent loops in the PFV intasome (Fig. 4f, bottom) and STC structures of other transposases and integrases have no contact with target DNA. Altogether, the unique aspects of PB target DNA recognition and integration are driven by the location of the active sites within the dimer, the interactions of the omega loops, and the relative positions of the target scissile bonds.

### *pB* transposition in cells

**Fig. 6:**
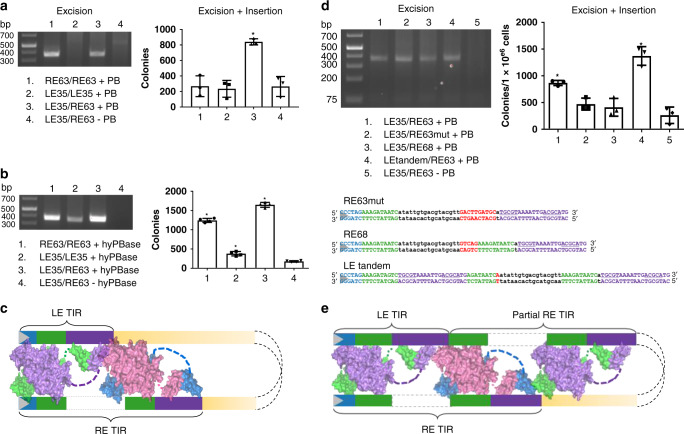
Proposed transpososome with LE/RE TIR bound and in vivo transposition assays. **a** Excision (left) and colony count transposition analysis (right) in cultured HT-1080 cells using wild type PB. Excision was assayed by PCR detection of repaired excision sites. Excision and subsequent integration are assessed by colony count, which represents events in which the resistance gene (located between *pB* TIRs) is integrated into the chromosome by *pB* transposition. The resulting cells form colonies in the presence of G418, indicating the full transposition pathway from excision to integration. Excision results are representative of three independent experiments. Colony counts, *n* = 3 independent experiments ± SD. **b** Excision (left) and Excision+integration (colony count) transposition analysis (right) in cultured HT-1080 cells using hyPBase. Excision and Excision+integration (colony count) transposition analysis in cultured HT-1080 cells is as in Fig. 6a. Colony counts, *n* = 4 independent experiments ± SD. **c** Proposed model for the asymmetrical binding of LE and RE TIRs by two PB dimers. Colored boxes in TIRs are the same as in Fig. 1b. **d** Excision and colony count transposition analysis in cultured HT-1080 cells of LE and RE TIR variants. RE63mut has a mutated green repeat, RE68 has five additional bp inserted as indicated in red, and LEtandem is lengthened by repeating a region of the RE TIR as shown in **e**. Excision assays shown are representative of three independent experiments. Colony counts, *n* = 3 independent experiments ± SD. **e** Proposed model representing assembly of PB with LEtandem and RE TIRs in a transpososome. The LEtandem TIR combines sequences of the LE TIR and RE TIR. The synaptic model includes three dimers of PB. Colored boxes in TIRs are the same as in Fig. 1b. Statistical analysis statement of **a**, **b**, and **d**: Data were analyzed using one-way ANOVA followed by Dunnett’s multiple comparison post-test comparing each column to the LE35/RE63 without control. All error bars show the standard deviation. Statistically significant differences were considered as follows: *p* ≥ 0.05 (ns) and *p* < 0.05 (*). Source data are provided as a Source Data file.

We used colony count and excision assays in cultured human cells using PB and *pB* transposon derivatives as a proxy for in vivo transposition^65^. Excision analysis uses PCR to amplify re-joined transposon plasmid ends recovered from transfected cells indicating transposon excision has occurred. The colony count assay involves excision of a neomycin resistance transposon (pTpB) from a transposon plasmid followed by integration into the genomes of cells. Cells that have undergone transposition grow and form colonies in the presence of G418 which allows selection for the neomycin resistance gene, thereby providing a quantitative readout of not only excision but also subsequent integration. To correlate with our structural data, we evaluated *pB* transposition using shortened TIRs of LE35/RE63. Experiments in HT-1080 cells transfected with *pB* transposon derivatives and a helper plasmid expressing PB indicated that only *pB* containing asymmetrical LE35/RE63 TIRs is active for both excision and integration; LE35/LE35 is inactive for both activities whereas RE63/RE63 can excise but not integrate using native PB (Fig. 6a). Evaluation of excision and integration with hyPBase^33^ revealed relaxed stringency for the LE35/RE63 pair as symmetrical RE63/RE63 was capable of both excision and integration whereas LE35/LE35 was also capable of excision and integration though to a lesser degree (Fig. 6b).

Several lines of evidence suggest that the PB synapse assembled in vivo is likely to consist of at least two dimers, perhaps bound to each other as a tetramer (modeled in Fig. 6c). The structures indicate that the CRDs form a dimer that binds the internal repeat (purple boxes in Fig. 6c) on only one end yet footprinting data indicates that the internal repeats on both the LE TIR and RE TIR are protected by PB^43^. Our in vitro integration assays with short oligonucleotide *pB* TIR substrates also provide indirect support for the notion of two dimer binding sites in the authentic PB synaptic assembly (Supplementary Fig. 1d). In these, RE33 was more active than RE63, the authentic TIR. RE33 may be an effective integration substrate because the insertion between RE bp 17–33 contains a sequence (TG**ACG**T**A**C**G**, bp 23–31) that resembles the second CRD-binding site on the LE between bp 27–35 (TG**ACG**C**A**T**G**). This cryptic CRD-binding site may be sufficient to allow the assembly of a RE33/RE33 complex analogous to that on LE35/LE35, a combination that is also active in vitro. The most reasonable explanation for the inhibition observed when RE33 is extended to RE63 is that bp 34–63 of the RE are almost identical to LE bp 7–35 (Fig. 1b); thus, under our assay conditions with limiting protein, they provide a competing binding site for a PB dimer. This is consistent with a second dimer binding site in the authentic LE/RE PB transpososome.

To probe if a higher order PB oligomer than a dimer is needed for activity in cells, we engineered a *pB* transposon in which we modified the TIRs in several ways. We hypothesized that inserting five bp of random sequence into the middle of the RE TIR (Fig. 6d, RE68) might disrupt any phased binding of two PB dimers. Indeed, when coupled with LE35, transposition activity decreased by ~50% (Fig. 6d, lane 3). A similar decrease is observed when the second green box in the RE TIR is randomized (RE63mut, Fig. 6d, lane 2), indicating that the specific sequence in this region is important. Finally, we modified *pB* by adding a third CRD-binding site on the LE (Fig. 6d, e, LEtandem), repeating the pattern of conserved regions as observed on the RE. When coupled with RE63, this modified *pB* transposon is ~two-fold more active for integration than the wild type LE35/RE63 combination (Fig. 6d, lane 4), suggesting that an additional dimer binding site stimulates activity, perhaps by promoting the formation of the synaptic complex.

## Discussion

The results here reveal that the distinguishing features of *pB* transposition, seamless excision and tetranucleotide targeting, are the consequences of a structural echo between flanking hairpin recognition and TTAA target recognition. The STC structure suggests that the reason *pB* almost invariably integrates at a TTAA tetranucleotide^44,66^ is a combination of structural features that include a dramatic target bend, a deformation that causes the TTAA to unpair, and a dense network of interactions that specifically recognize TTAA in single-stranded form. It may be that PB identifies its specific target site by randomly sampling DNA sequences until it encounters one that can be bent enough to unpair its center four bps and that it can subsequently stabilize in ssDNA form.

Strand transfer occurs across the minor groove, in contrast with other known transpososomes or intasomes^40,49,56,60–64,67^(Supplementary Fig. 8), and the location of active sites in the dimer mandates the 4-bp offset in transesterification positions (Fig. 4e). After specific integration and repair, *pB* is therefore flanked by 4-bp TSDs with a TTAA sequence (Fig. 1a). Crucially, only a subset of the interactions that recognize the single-stranded TTAA target is used to bind and stabilize the hairpin intermediate that form on the transposon ends upon generation of the DSB. At excision, the transposase does not require a specific flanking sequence^44^, but it does ensure that a hairpin is formed. However, if the flanking sequence is a TTAA, which is the TSD from the previous transposition, upon excision, this will generate the 4-nt TTAA hairpins on the transposon ends. This chain of structurally linked reactions is what leaves behind complementary 4-nt overhangs at the site of genomic excision and allows for seamless repair. Other transposons do not exhibit seamless excision because there is no coupling between how the DSBs are generated at transposon ends during excision and how target is recognized. For example, the *SB* transposon is excised with 5′ recesses because cleavage on the NTS is 3-nt within the transposon itself. The resulting 3-nt CAG overhangs at the donor site are not complementary and when the DSB is repaired, an excision footprint is left that also includes one extra copy of the TA target site of *SB*^9^. The plasticity of the flanking sequence at excision is beneficial for the life cycle of a *pB* transposon as it allows for mobility even if the flanking sequence is not TTAA for any reason. Otherwise, the transposon would become trapped and this would spell the end of its life as a mobile element.

The structural view of *pB* transposition is consistent with data in cells, where TTAA target recognition and integration are more stringently controlled by *pB* than in in vitro reactions involving hairpin formation or hydrolysis. This is evident from observations that cellular integration occurs overwhelmingly at TTAA sites^44,66^. This most likely reflects a requirement for the hydrogen bond between T0_T_ and A3_T_ that cannot be satisfied if T0_T_ is substituted by any other nucleotide. In contrast, at the excision step of mobilization, *pB* does not require that its flanking sequence be TTAA; non-TTAA flanked versions are integrated with comparable efficiency relative to WT, but the TTAA targeting specificity remains^44^. Also, as shown here (Fig. 6a), *pB* variants such as RE63/RE63 can be excised but not integrated, indicating that excision is more permissive than integration.

The *pB* from *T. ni* is but one member of a larger superfamily of *pB*-like elements^68^. The superfamily includes *piggyBat*, one of the very few DNA transposons natively active in vertebrates^69,70^; domesticated elements such as *PiggyMac* which play a pivotal role in ciliate nuclear development^71^; and the larger group of *piggyBac-derived* (PGBD) proteins^68^. In humans, PGBD proteins have been implicated in premature ovarian failure^72^ and childhood tumor development^73^, and the structures here will provide insight into their action.

A popular version of PB in genomic applications is hyperactive PB, hyPBase, that has seven amino acid substitutions relative to *T. ni* PB, and the structures suggests how they may stimulate activity. As two (I30V, S103P) are in the disordered N-terminus, this region is clearly important but most likely not for chemical catalysis; it will be interesting to determine if this region is involved in PB–PB interactions or interactions with cellular factors that contribute to transposition. G165S, located close to the donor DNA, presumably forms an additional interaction with it; M282V is in the omega-loop which may allow better stacking of Y283 with A3_T_ of the target sequence. S509G is at the kink of an α-helix (α12) that interacts with both LE DNAs, and the increased flexibility afforded by the G mutation may strengthen DNA binding. While N538K is the last residue visible in the DDBD before the disordered loop that connects to the CRD, the loop itself is very negatively charged (E539, D545, E549, E550) and the K mutation might provide a stabilizing effect. N571S appears well-positioned to interact favorably with a phosphate at the CRD-binding site. Finally, the phenotype of the R372A/K375A mutant, proficient in excision but defective in integration (Exc^+^Int^−^), is most likely due to the loss or weakening of flanking target DNA binding as both these residues of the insertion domain interact with target. Curiously, two of these amino acid substitutions (G165S and M282V) are naturally present in PiggyBat^69^ and I30 is similarly substituted with a smaller hydrophobic residue, leucine.

An enigmatic feature of *pB* is the asymmetric TIR organization that is required for transposition activity within cells, with a minimal functional version consisting of LE35 and RE63 (Fig. 1b). In the SNHP and STC complexes assembled with symmetrical LE35/LE35 TIRs, we observed that a CRD dimer binds to only one of the TIRs. The structures suggest that a CRD monomer is not sufficient to bind and that CRD dimerization is coupled to palindromic internal repeat binding, probably allosterically resulting in DNA bending. When taken together, our data point to a model in which, in cells, a higher order PB assembly synapses a LE/RE TIR pair with a catalytic PB dimer at the transposon tip (as seen in the structures here) and a second inner PB dimer bound predominantly through interactions with the RE internal repeat sequence (Fig. 6c). It is possible that the disordered negatively charged N-terminal region of PB (1–117) could assist in higher-order assembly as it would be in the appropriate position extending from the inner dimer to interact with the CRD dimer bound to the LE through electrostatic interactions^74^. Such a model also implies that the inner dimer is not catalytic.

With hyPBase, there is some detectable activity with LE/LE TIRs, suggesting that a catalytic PB dimer alone can function if the system is pushed. One fundamental difference between transposition experiments in vitro and in cells is the vast amount of DNA in the nucleus of a cell. It is possible that a higher order assembly is required to provide enough binding strength so that *pB* LEs and REs present in cells can be brought into each other’s physical proximity to form an effective synapse in the background of a large amount of other DNA. This raised the intriguing possibility that *pB* transposition in cells might be limited at the synapse step. To test this, we designed a modified LE TIR (LEtandem) by adding additional *pB*-binding sites. Indeed, LEtandem in combination with the RE TIR showed increased transposition activity in cells (Fig. 6d), mostly likely by directing the assembly of an even higher order transpososome. This tandem TIR also suggests a strategy for designing improved *pB* vectors for gene delivery. A similar approach has been tried with *SB*^75^ with success. However, as the *SB* transpososome structure is not known, the binding sites were simply duplicated and a mutation to prevent cleavage had to be introduced. The requirement for large multimeric assemblies of transposases and integrases for in cell activity while only a dimeric subset is used for cleavage and strand transfer steps is a common feature of many of the systems studied^56,63,76^.

**Fig. 7:**
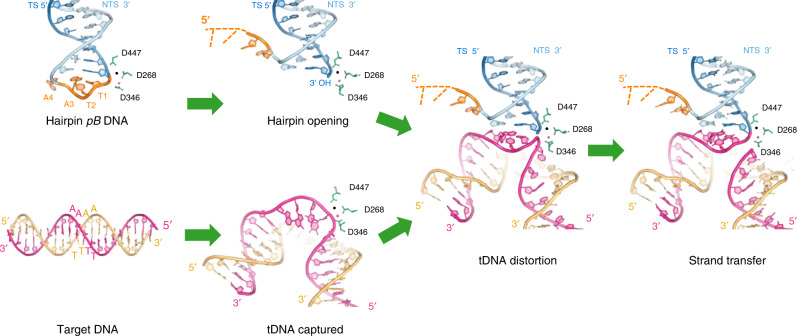
Model of *piggyBac* transposition. Upon liberating the transposon from the donor site, PB then catalyzes hairpin DNA opening and integration into a 4-bp TTAA target site. Target DNA is accessed through the minor groove and undergoes a severe bend and distortion. Hairpin opening and target DNA capture may or may not be concerted at the active site. The observed metal ion is shown as a red sphere and a second metal ion as a black sphere. Catalytic residues are shown as green sticks. DNA molecules are colored as in Fig. 1d. NTS non-transferred strand, TS transferred strand.

The SNHP and STC structures here, which have captured two snapshots along the *pB* transposition pathway (Fig. 7), reveal the remarkable way that *pB* achieves seamless excision and its strict TTAA target site specificity. They serve as a foundation for future development of improved genetic tools.

## Methods

### Protein expression and purification

The gene for full-length *Trichoplusia ni* (*T. ni*) PB transposase was codon-optimized for mammalian expression (IDT) and cloned into the pD2610 expression vector (a gift from Dr. Alex Kentsis from Memorial Sloan Kettering Cancer Center) between *Bam*HI and *Kpn*I restriction sites, downstream of an N-terminal maltose-binding protein (MBP) tag followed by a TEV protease cleavage site. The plasmid pD2610-MPB-PB was transfected into 500 ml EXPI293F cells (Thermo Fisher Scientific) for transient protein expression using a standard PEI transfection protocol. The transfected cells were supplied with 500 ml Expi293 expression medium after 24 h. Cells were harvested 3 days after transfection at 300 × *g* and stored at −80 °C.

Cells expressing MBP-tagged PB were resuspended in lysis buffer containing 25 mM Tris–Cl, pH 7.5, 500 mM NaCl, 1 mM TCEP, and protease inhibitor cocktail (Roche). The cells were lysed by three cycles of sonication. Cell lysates were centrifuged at ~95,000 × *g* for 30 min at 4 °C (Beckman Coulter Optima L-100 XP Ultracentrifuge, Type 45 Ti rotor). The supernatant was filtered and mixed with 10 ml amylose resin (New England BioLabs) equilibrated with lysis buffer. After one hour of continual rotation, the mixture was loaded onto a gravity flow column and washed with 100 ml lysis buffer. The protein was eluted with 50 ml elution buffer (25 mM Tris–Cl, pH 7.5, 500 mM NaCl, 10 mM maltose, 1 mM TCEP, and protease inhibitor cocktail). The eluate was incubated with TEV protease and dialyzed against dialysis buffer (50 mM Tris–Cl, pH 7.5, 500 mM NaCl, and 1 mM TCEP) for 16–20 h at 4 °C. The cleaved MBP tag and TEV protease were separated from PB using a 5 ml HiTrap Heparin HP column (GE Healthcare) with a linear-gradient elution from 500 mM to 1 M NaCl. PB was further purified by SEC on a Superdex 200 HiLoad 16/60 column in 50 mM Tris–Cl, pH 7.5, 500 mM NaCl, and 0.5 mM TCEP. Purified PB was concentrated to about 30 mg/ml and stored at −80 °C. The yield is about 3 mg/l cell culture.

### SEC–MALS analysis of full length PB protein

For determination of molecular mass by SEC coupled with multi-angle light scattering (SEC–MALS) analysis, purified PB at 4 and 2 mg/ml was applied to a Superdex 200 Increase 10/300 (GE Healthcare) column in 20 mM HEPES pH 7.5, 100 mM NaCl, and 1 mM TCEP. The column eluent was passed through a 660 nm photometer and a refractometer with a dynamic light scattering attachment (Wyatt Technology). Analysis was performed using ASTRA version 7.1.2.5 software and yielded a single molar mass of 135,700 g/mol, consistent with a dimer. The SEC–MALS was performed in the Biophysics Core Facility at NHLBI/NIH.

### SNHP and STC complex assembly

The SNHP was prepared by mixing equimolar amounts of PB protein and LE hairpin DNA, and incubating for 1 h at room temperature (25 °C), followed by SEC purification using a Superdex 200 Increase 10/300 column (GE Healthcare) in 50 mM Tris–Cl, pH 8.0, 100 mM NaCl, 5 mM CaCl_2_, and 1 mM TCEP. The LE hairpin DNA was prepared by heating LE35hrpn (5′- CATGCGTCAATTTTACGCAGACTATCTTTCTAGGGTTAACCCTAGAAAGATAGTCTGCGTAAAATTGACGCATG -3′; see also Supplementary Fig. 2a) at 80 °C for 10 min and immediately transferring to an ice bath. To confirm hairpin DNA opening activity, SEC fractions were supplied with MgCl_2_ and incubated at 25 °C for 1 h then loaded to a denaturing 15% TBE–urea gel for detection in addition to the SDS–PAGE analysis (Supplementary Fig. 2b).

The strand transfer complex (STC) was reconstituted by mixing PB protein and LE STC DNA in a 1:2 molar ratio in assembly buffer (50 mM Tris–Cl, pH 8.0, 500 mM NaCl, 10 mM CaCl_2_, and 1 mM TCEP) and dialyzing to a final buffer (50 mM Tris–Cl, pH 8.0, 200 mM NaCl, 10 mM CaCl_2_, and 1 mM TCEP) at 4 °C overnight. The LE STC DNA was generated by annealing equimolar amounts of four oligonucleotides: (1) 5′-CCGGCCCTAGAAAGATAGTCTGCGTAAAATTGACGCATGCA-3′; (2) 5′-TGCATGCGTCAATTTTACGC-3′; (3) 5′-AGACTATCTTTCTAGGGTTAAGACTGTGCCGC-3′; (4) 5′-GCGGCACAGTC-3′ (see also Supplementary Fig. 3a). The complex was purified by SEC using a Superdex 200 Increase 10/300 column (GE Healthcare). After purification, the fractions were analyzed by both SDS–PAGE and denaturing gel (Supplementary Fig. 3b).

### Cryo-EM sample preparation and data collection

To prepare grids for cryo-EM data collection, purified samples (3 μl at 0.8 mg/ml) were applied to freshly glow-discharged C-FLAT holey carbon grids (CF-1.2/1.3-3Au, Electron Microscopy Sciences). They were then blotted inside a Vitrobot Mark IV (FEI company) chamber using 4-s blotting time with 100% relative humidity at room temperature, and plunge-frozen in liquid ethane cooled by liquid nitrogen. Cryo-EM images were collected on a Titan Krios electron microscope (Multi-Institute Cryo-EM Facility (MICEF), NIDDK, NIH) operated at 300 kV equipped with a Gatan K2 Summit direct electron detector camera. Movies were recorded using super-resolution counting mode at a nominal magnification of 130,000×, corresponding to a calibrated super resolution pixel size of 0.53 Å per pixel on the specimen. The defocus values ranged from −1.0 to −2.0 μm. The cryo-EM movies were recorded using a semi-automated program SerialEM^77^. For the SNHP data set, the dose rate on the camera was set to be about 8.6 e^−^ per physical pixel per second. The total exposure time for each movie was 10 s with a total exposure dose of 73.7 e^−^/Å^2^ on the specimen. Each movie was fractionated into 50 frames, with 0.2 s per frame. For the STC data set, the dose rate was about 8.73 e^−^ per physical pixel per second. The total exposure time for each movie was 6 s with a total exposure dose of 46.6 e^−^/Å^2^. Each movie was fractionated into 30 frames. The statistics of cryo-EM data collection are summarized in Supplementary Table 1.

### Cryo-EM data processing

Unless stated otherwise, all image processing was performed using the RELION 3.0 package^78^ at the NIH HPC Biowulf cluster (http://hpc.nih.gov). A total of 5686 and 9243 movies were collected for the SNHP complex and STC, respectively. MotionCor2 1.1.0^79^ was used for beam-induced motion correction and dose weighting. The output aligned micrographs were binned 2× in Fourier space, resulting in a pixel size of 1.06 Å for further processing. Micrographs were then visually screened and poor-quality ones (too few good particles, too much contamination, abnormal Fourier patterns, or too much aggregation) were discarded. The non-dose-weighted micrographs were used for contrast transfer function (CTF) parameters estimation using Gctf 1.06^80^. The dose-weighted micrographs were used for automated particle picking, classification, and map reconstruction.

For the SNHP dataset, about 2.6 million particles were picked automatically from 3855 micrographs by crYOLO^81^. The auto-picked particles were subjected to 2D classification to remove bad particles. An 3D initial model was generated from the cleaned particle stack and used as a starting reference for 3D classification without imposing symmetry. The best class showing high-resolution features was used for gold-standard refinement, followed by per-particle CTF refinement and Bayesian polishing. The map generated from these polished particles had good density at the catalytic domain but poorly interpretable density at the CRD. To better resolve the CRD domain, we performed an additional masked 3D classification using a mask covering only the CRD domains. After classification into 10 classes, one class (35,960 particles) that showed significantly better CRD density was further improved by a second round of CTF refinement and Bayesian polishing. The final resolution was 3.66 Å and the map showed improved density in the catalytic domains as well (Supplementary Fig. 4a).

For the STC dataset, about 6.7 million particles were picked from 6738 micrographs by crYOLO^81^. These particles were cleaned by rounds of 2D and 3D classifications, resulting in 372,228 good particles followed by per-particle CTF refinement and Bayesian polishing. These polished particles were then subjected to masked 3D classification into six classes using a soft mask covering the whole STC; we picked two of these with the best density at the CRD. The 43,512 particles that belonged to these classes were used for a second round of per-particle CTF refinement and Bayesian polishing. A final masked 3D refinement generated a 3.47 Å map (Supplementary Fig. 4b).

All refinements followed the gold-standard refinement procedure, in which two half datasets were refined independently. The overall resolution was estimated based on the Fourier Shell Correlation (FSC) threshold at 0.143 between the two half-maps, after a soft mask was applied to mask out the solvent region. The local resolutions were estimated from two unfiltered half maps, using LocalRes within RELION 3.0^78^. Map visualization was carried out in UCSF Chimera^82^. All figures were prepared using either PyMol (http://www.pymol.org) or ChimeraX^83^.

### Ab initio model building and refinement

Except the CRD, no homology model was available to be fitted into the density maps. Both SNHP and STC cryo-EM density maps were of sufficient quality for ab initio atomic model building since distinct structural features (backbones of protein and DNA, secondary structure elements, and large sidechains) appeared in the maps, although the CRD density was not as well resolved as other regions of the complex. We generated a sharpened map for the SNHP in Phenix^45^ and build the initial model with map_to_model also in Phenix. The output model was examined in COOT^84^ and the sequence was assigned based on visible side chain densities. The bound DNA was built manually. The NMR model of the CRD structure (PDB 5LME) was docked into the density as a rigid body, followed by manual adjustment in COOT. The linker between the CRD and catalytic domain had no interpretable density and was built as a coil using COOT. The model was rebuilt with Rosetta’s rebuilding tools^47^ using Rosetta’s Cartesian Sampler generating 200 decoys. Execution was parallelized by GNU parallel^85^. Out of these, the five lowest Rosetta energy decoys were displayed in O^86^ and a consensus model that best fit the density was generated manually. This model was refined by the Relax protocol in Rosetta with tightly restrained *B* factor optimization.

For the STC, the initial model was built from the SNHP complex and fitted into the density with Coot. Real-space refinement against the cryo-EM density map was carried out by the Rosetta Relax protocol including tightly restrained *B* factor refinement. The validation check for model stereochemistry of both complexes was performed in MolProbity^87^ (Supplementary Table 1).

### In vitro transposition assay using TIR DNA

Purified PB (200 nM) was incubated with SC 100 ng pUC19 plasmid and 100 nM TIR DNA in reaction buffer containing 25 mM Tris–Cl pH 8.0, 25 mM NaCl, 5 mM MgCl_2_, 2 mM DTT, 1X BSA (NEB) (final volume 100 μl). The mixture was incubated at 30 °C for 1 h unless otherwise indicated. The reaction was stopped by adding 5 μl 0.5 M EDTA, 2 μl proteinase K (800 U/ml, NEB) followed by incubation at 37 °C for 30 min. DNA was extracted for analysis by ethanol precipitation, dissolved in 10 μl 10 mM Tris–Cl pH 8.0 solution followed by electrophoresis on 1.5% agarose gel running in 1X TAE buffer at 100 V for 70 min. Gels were stained with ethidium bromide for 20 min and imaged using a ChemiImager 5500 gel imaging system (Alpha Innotech).

To generate linearized pUC19 as target DNA, SC pUC19 was treated by *Xma* I (a single cutter) and re-purified. Purified PB (200 nM) was incubated with 100 ng linearized pUC19 plasmid and 100 nM RE TIR DNA variants. The reactions were incubated overnight at 30 °C. Other steps are as described above.

### In vitro transposition assays using a mini-transposon

To generate a mini-transposon, we first cloned the *pB* LE35 and RE63 into pHL2577^88^ using the QuikChange method, and then used the plasmid (called pBLE35RE63) as a template to generate the mini-transposon by PCR. After purification and extraction from an agarose gel, we incubated the 2.9 kb mini-transposon in which the Kan^r^ gene was flanked by LE35 and RE63 with purified PB, pUC19 in the above buffer overnight at 30 °C. The ethanol-precipitated DNA was transformed into TOP10 cells and colonies were selected by Amp+Kan+ agar plates. The plasmids were extracted and sequenced using primers (5′-CTGAGTGCGCAGAACGACACG-3′ and 5′-CCGGCGGGGACGAGGCAAGC-3′) for sequences inside the mini-transposon to detect the TSD sites generated by transposition^89^.

### In vitro DNA cleavage and hairpin DNA formation assay

Purified PB (200 nM) was incubated with FAM-labeled DNA substrates (5 nM) in reaction buffer (25 mM Tris–Cl pH 8.0, 25 mM NaCl, 5 mM MgCl_2_ or CaCl_2_, 1x BSA, 5% glycerol and 2 mM DTT; 50 μl final reaction volume) at 30 °C for 1 h. The reaction was stopped by adding 12.5 μl 98% formamide. Reactions were loaded on a denaturing 15% TBE-urea gel, followed by electrophoresis at 250 V for 25 min at room temperature in 1X TBE buffer. The gel was imaged using a Typhoon FLA 9500 (GE Healthcare).

### In cell excision and transposition assays

pCMV-HA-PB^90^ encodes a hemagglutin (HA)-tagged PB transposase. All transposon plasmids were derived from pTpB^65^ using standard molecular biology techniques. All plasmids were confirmed by DNA sequencing. For excision assays, 3 million HT-1080 cells were seeded into a 100 mm dish. The next day, cells were transfected with 10 µg of the transposon and 5 µg transposase plasmids using lipofectamine (ThermoFisher). One day later, cells were trypsinized and excision analysis PCR was performed using the following primers (forward: 5′-ATGCGGCATCAGAGCAGATT-3′, reverse: 5′-TGTGTGGAATTGTGAGCGGA-3′)^65^. For colony counts, 0.4 million HT-1080 cells were seeded into each well of a six-well plate. The next day, cells were transfected with 1 µg of transposon and 0.5 µg of transposase plasmids using lipofectamine. One day later, cells were trypsinized and diluted to 100 mm dishes followed by selection with 600 µg/ml of the antibiotic G418 for 8–10 days. Colonies of cells were fixed in 10% formaldehyde/phosphate-buffered saline (PBS), stained with 1% methylene blue in PBS, and counted^91^.

### Reporting summary

Further information on research design is available in the Nature Research Reporting Summary linked to this article.

## Supplementary information

Supplementary Information

Peer Review

Reporting Summary

## Data Availability

The data that support this study are available from the corresponding author upon reasonable request. The atomic coordinates of SNHP and STC have been deposited in the protein data bank (PDB) with the accession codes: 6X67 for the STC, and 6X68 for the SNHP complex. The EM maps have been deposited in the Electron Microscopy Data Bank (EMDB) with accession codes: EMD-22072 for the STC and EMD-22073 for the SNHP complex. The source data underling Fig. 6 and Supplementary Figs. 1, 2, and 3 are provided as a Source Data file.

## Source data

Source Data
