# Supplementary material for: Structural basis of seamless excision and specific targeting by piggyBac transposase
Source: Nat Commun. 2020 Jul 10;11:3446. doi: 10.1038/s41467-020-17128-1 (PMC7351741; doi:10.1038/s41467-020-17128-1)
Supplement: Supplementary file 1 — Supplementary Information [file 41467_2020_17128_MOESM1_ESM.pdf]

Supplementary Information for:

# Structural basis of seamless excision and specific targeting by *piggyBac* transposase

Qiujia Chen[1], Wentian Luo[2,3], Ruth Ann Veach[2,3], Alison B. Hickman[1], Matthew H. Wilson[2,3,4] and Fred Dyda[1]*

[1]Laboratory of Molecular Biology, National Institute of Diabetes and Digestive and Kidney Diseases, National Institutes of Health, Bethesda, MD, 20892 USA

[2]Department of Medicine, Division and Nephrology and Hypertension, Vanderbilt University Medical Center, Nashville, TN, USA; [3]Department of Veterans Affairs, Nashville, TN, USA; [4]Department of Pharmacology, Vanderbilt University, Nashville, TN, USA

*Correspondence: fred.dyda@nih.gov

This document includes Supplementary Figures 1-8, Supplementary Figure Legends.

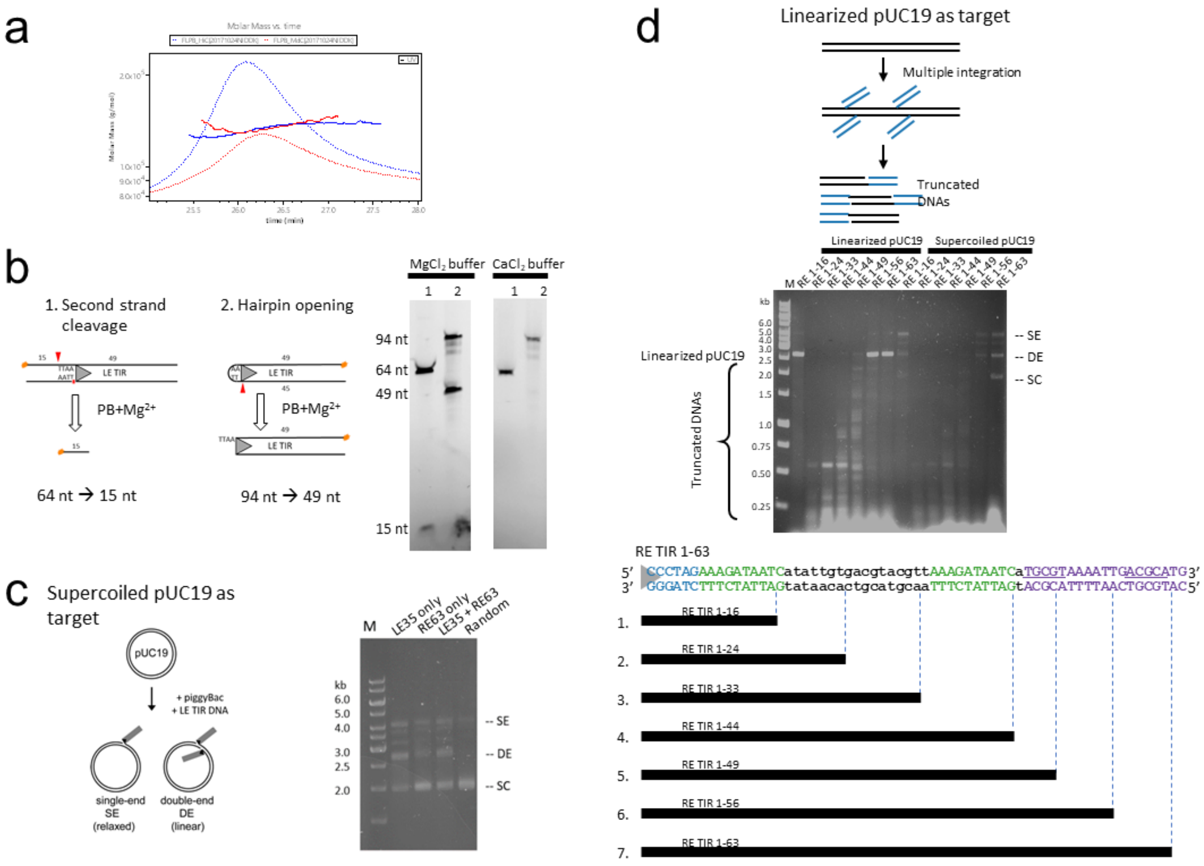


**Supplementary Figure 1. Biochemical data.**

**a.** Size-exclusion chromatography coupled with multi-angle light scattering (SEC-MALS) indicated that PB is a dimer in solution. The blue and red curves correspond to protein concentration at 4 mg/ml and 2 mg/ml, respectively. SEC-MALS was run on a Superdex 200 Increase 10/300 column.

**b.** DNA substrates labeled with the fluorescent probe FAM (orange stars) were used to detect DNA cleavage activities of PB. 200 nM purified PB protein and 5 nM FAM-labeled DNA substrates were incubated at 30 °C for 1 hour with either 5 mM $MgCl_2$ or $CaCl_2$, and the substrate and product DNA strands separated using denaturing 15% TBE-urea gels. Diagram 1 on the left shows the conversion of the bottom strand pre-nicked transposon end to a hairpin, in which a 15 nt FAM-labeled DNA is generated from the 64 nt top strand. When the hairpin DNA is opened (diagram 2 on right), the 94 nt DNA substrate becomes 49 nt. Red triangles indicate the cleavage sites. The result represents one of three independent experiments. Source data are provided as a Source Data file.

**c.** Transposition assay using supercoiled (SC) pUC19 as target DNA. Blunt-ended LE TIR or RE TIR were used as pre-cleaved transposon ends. Relaxed and linear plasmids representing single-end (SE) and double-end (DE) products, respectively, were separated using a 1.5% agarose gel. In the assay, 200 nM PB protein was incubated with 100 ng pUC19 plasmid and 100 nM TIR DNA in the presence of 5 mM $MgCl_2$. For LE35+RE63, 50 nM of each was mixed in the reaction. The result represents one of three independent experiments. Source data are provided as a Source Data file.

**d.** Transposition assay using different lengths of RE TIR and either linearized or supercoiled pUC19. Reactions (200 nM PB protein, 100 ng pUC19 plasmid and 100 nM RE TIR DNA variants) were incubated overnight at 30 °C. The smeared DNA on the bottom is a mixture of small truncated DNA resulting from pUC19 cleavage and the free RE TIR substrate. Note that RE 1-16, RE 1-56 and RE 1-63 have almost no detectable activities. However, when the length of the RE TIR was gradually shortened from RE 1-56 to RE 1-24, increasingly more pUC19 DNA was transformed into truncated DNA. The result represents one of three independent experiments. Source data are provided as a Source Data file.

a

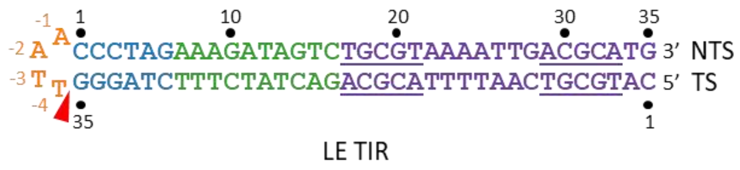


b

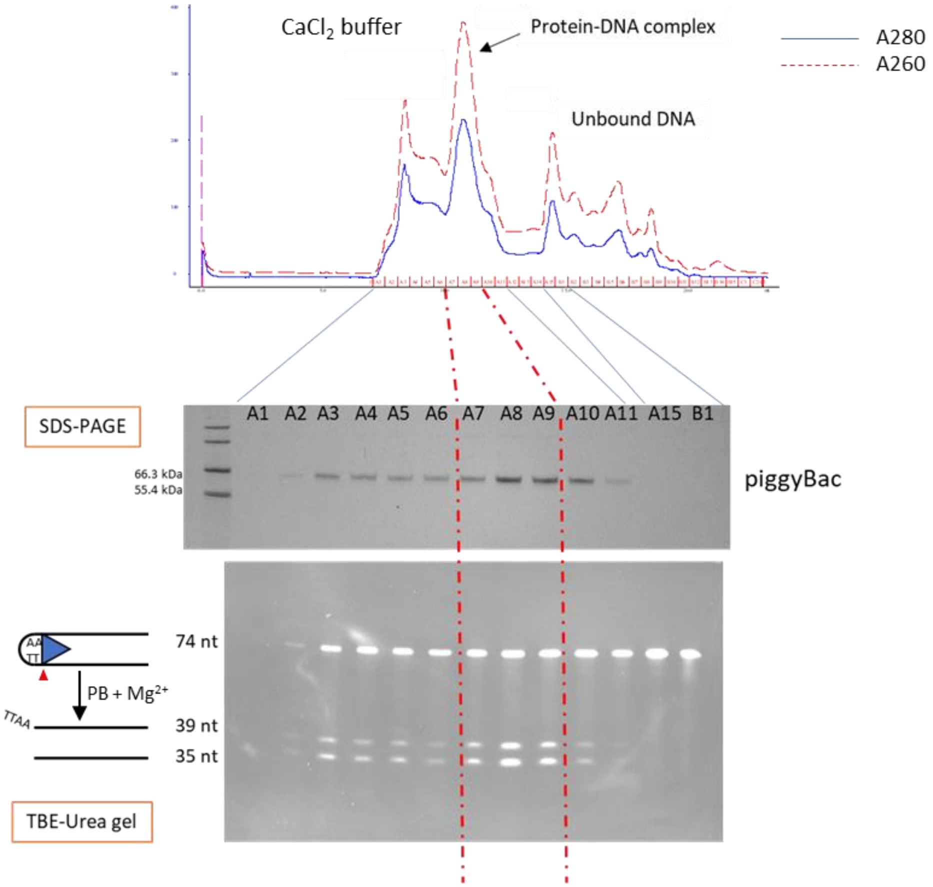


**Supplementary Figure 2. SNHP complex assembly.**

**a.** DNA used in the SNHP structure. The hairpin DNA contains LE TIR and donor flanking TTAA (orange). NTS: non-transferred strand. TS: transferred strand. A red triangle indicates the hairpin opening cleavage site. The color scheme is defined in Fig. 1b,d.

**b.** PB and LE-TIR hairpin DNA (74 nt) were assembled in $CaCl_2$ buffer and assessed by size exclusion chromatography. Fractions were analyzed with SDS-PAGE (top) to identify those in which the piggyBac protein was present. To confirm that the complexes were catalytically-active, samples of each fraction were supplied with 10 mM $MgCl_2$ and incubated at 30 °C for 30 min. The expected products of hairpin-opening (35 nt and 39 nt) were detected using 15% TBE-urea (bottom). The result represents one of three independent experiments. Source data are provided as a Source Data file.

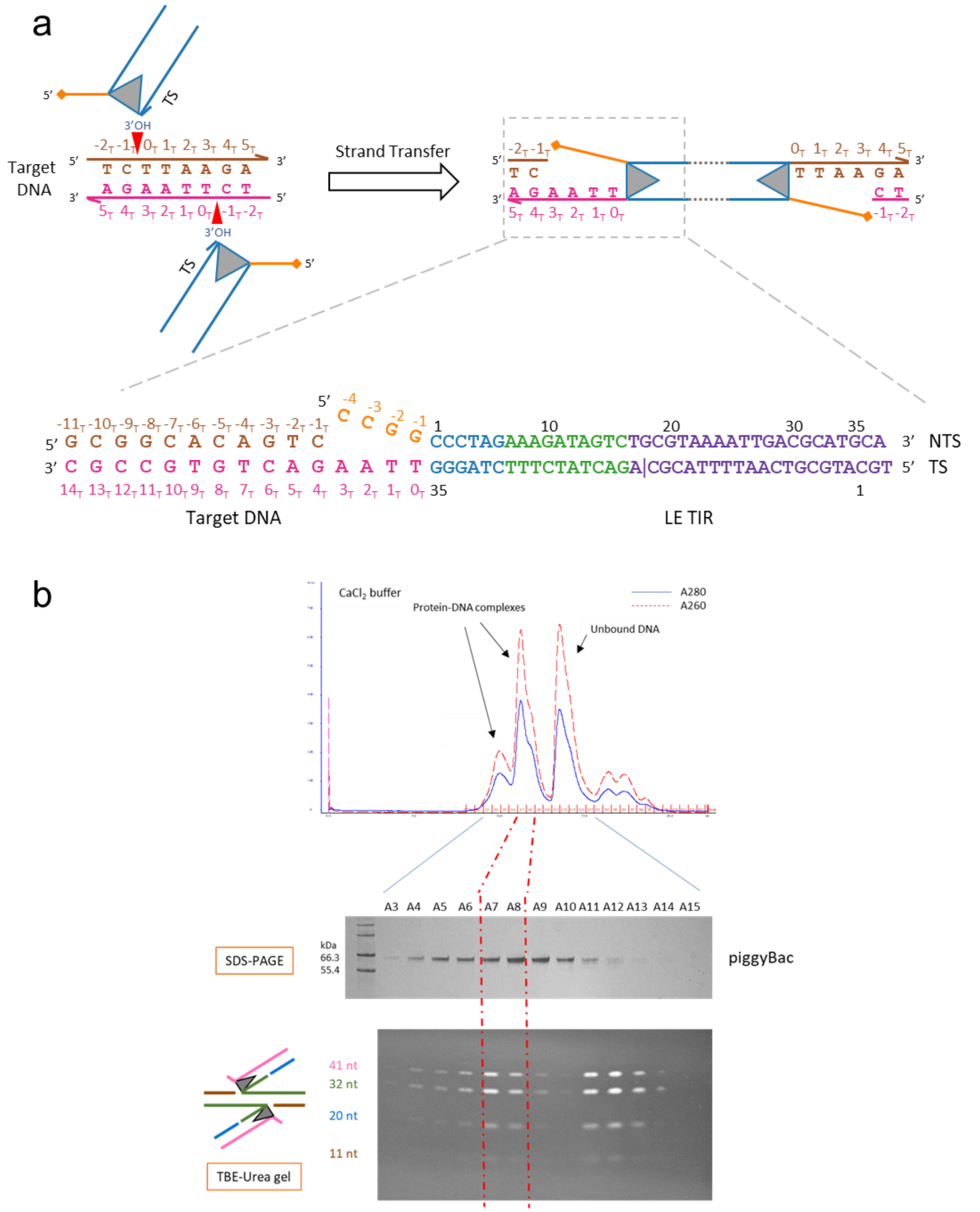


**Supplementary Figure 3. STC complex assembly.**

**a.** DNA used in the STC structure. The TS strand covalently connects to a target DNA (pink) that contains the TTAA target site. Target DNA has 11 bp of flanking DNA. The flap TTAA of the donor DNA (orange) was replaced with CCGG to prevent self-complementary base-pairing with the target site. STC DNA was annealed using four oligonucleotides, with a break in the TS indicated by the bar. The color scheme is defined in Fig. 1b,d.

**b.** PB and STC DNA were assembled in $CaCl_2$ buffer and assessed by size exclusion chromatography. STC DNA was annealed using four DNA fragments (11 nt, 20 nt, 32 nt and 41 nt) as shown in (a). Protein was detected by SDS-PAGE (top) and STC DNA using 15% TBE-urea denaturing gels (bottom). The result represents one of three independent experiments. Source data are provided as a Source Data file.

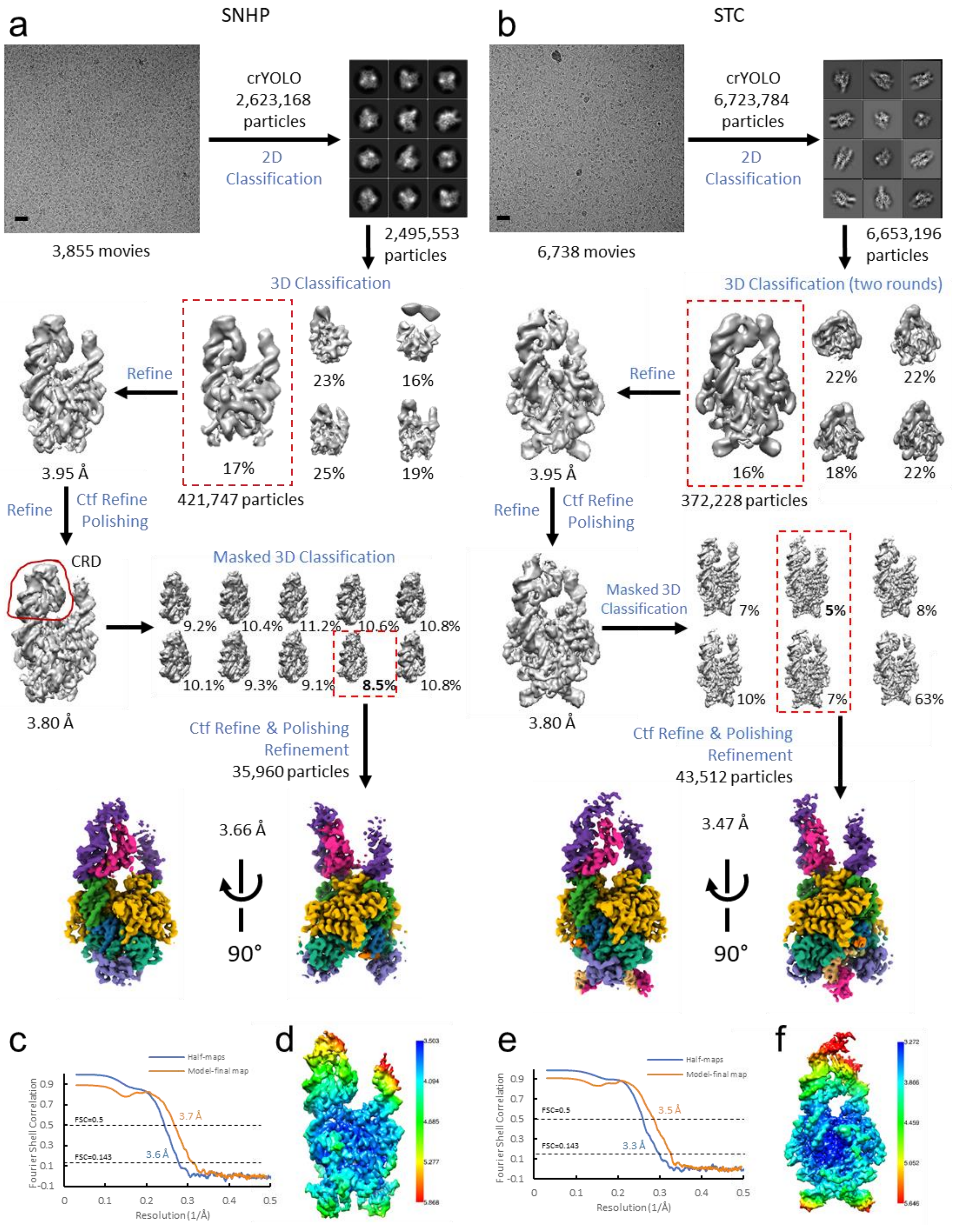


**Supplementary Figure 4. Cryo-EM data processing of the SNHP and STC complexes.**

**a.** Flowchart of SNHP data processing. The cryo-EM data were processed in RELION 3.0. The final map was generated using UCSF ChimeraX, with color scheme as defined in Fig. 1d. The

micrograph represents one of 3855 independent movies from the dataset. The scale bar is 50nm in the micrograph.

**b.** Flowchart of STC data processing. The cryo-EM data were processed in RELION 3.0. The final map was generated using UCSF ChimeraX, with color scheme as defined in Fig. 1d. The micrograph represents one of 6738 independent movies from the dataset. The scale bar is 50nm in the micrograph.

**c.** Half-maps FSC and model-final map FSC curves of SNHP. They were generated from ValidationCryoEM of the phenix suite.

**d.** Local resolution of the SNHP reconstruction generated by LocalRas in RELION 3.0.

**e.** Half-maps FSC and model-final map FSC curves of STC.

**f.** Local resolution of the STC reconstruction.

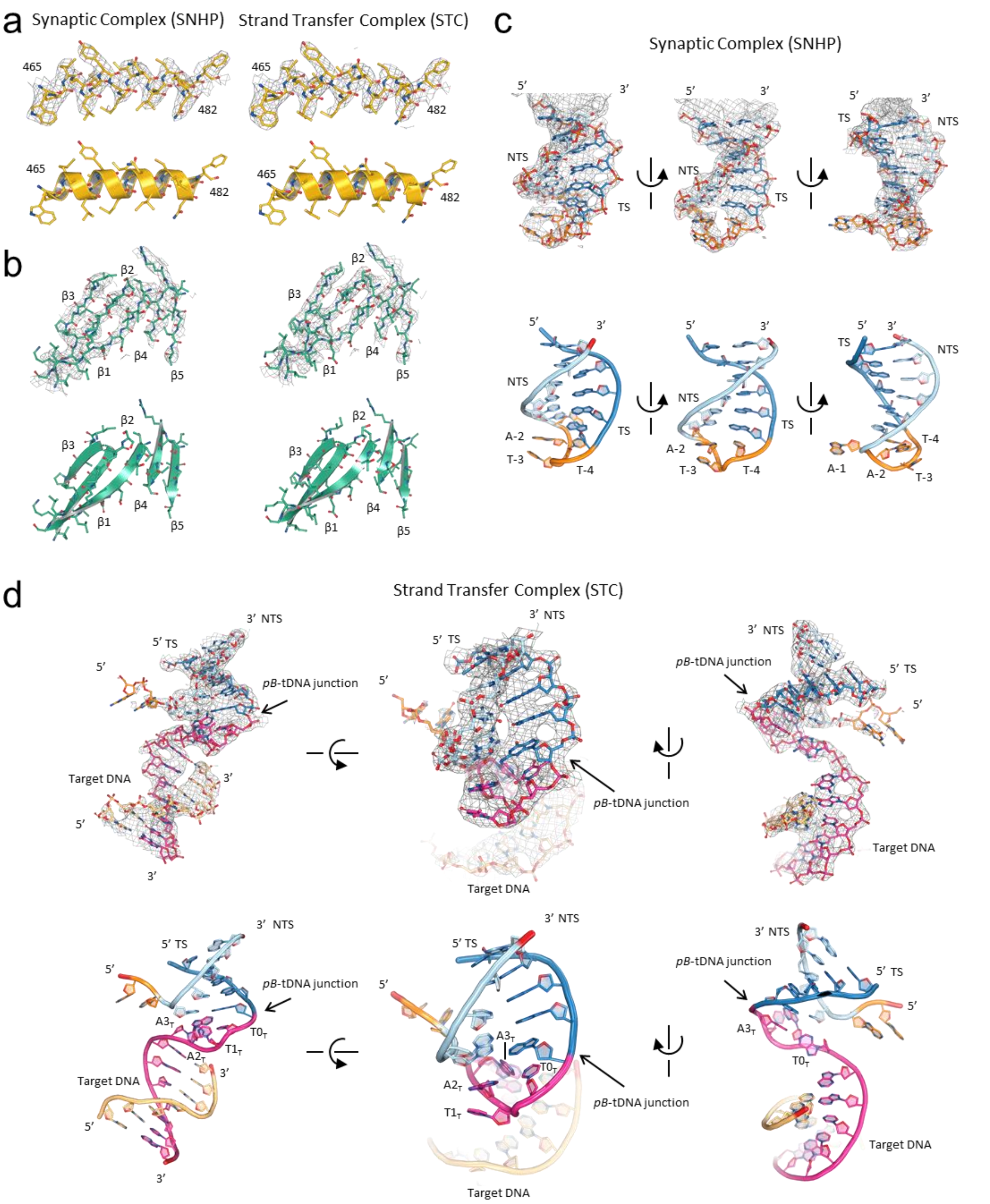


**Supplementary Figure 5. Representative regions of Cryo-EM densities superimposed on the atomic models.**

**a.** Representative α-helices (residues 465-482) from both complexes.

**b.** Five β-strands in the RNaseH-like folds from both complexes.

**c.** The hairpin DNA substrate in the SNHP complex.

**d.** The DNA model in the STC complex.

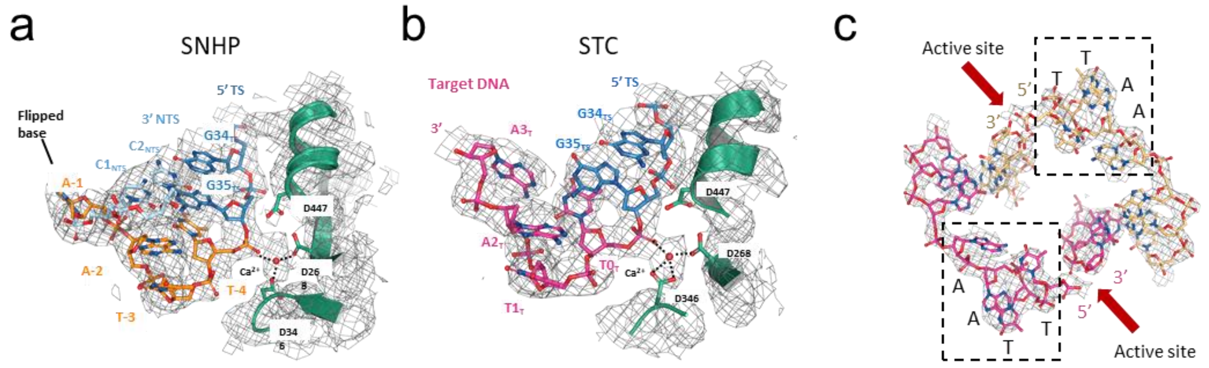


**Supplementary Figure 6. Representative regions of Cryo-EM densities**

**a** and **b.** The active sites of the SNHP and STC with the corresponding cryo-EM maps (gray meshes) superposed. Bound $Ca^{2+}$ is shown as a red sphere. Catalytic residues are shown as green sticks. DNA is rendered as sticks and molecules are colored and numbered as in Fig. 4a. NTS, non-transferred strand; TS, transferred strand.

**c.** View of the TTAA in the target DNA, superposed with the cryo-EM map (gray mesh). The target site is unpaired. Red arrows indicate the relative orientation of PB active sites.

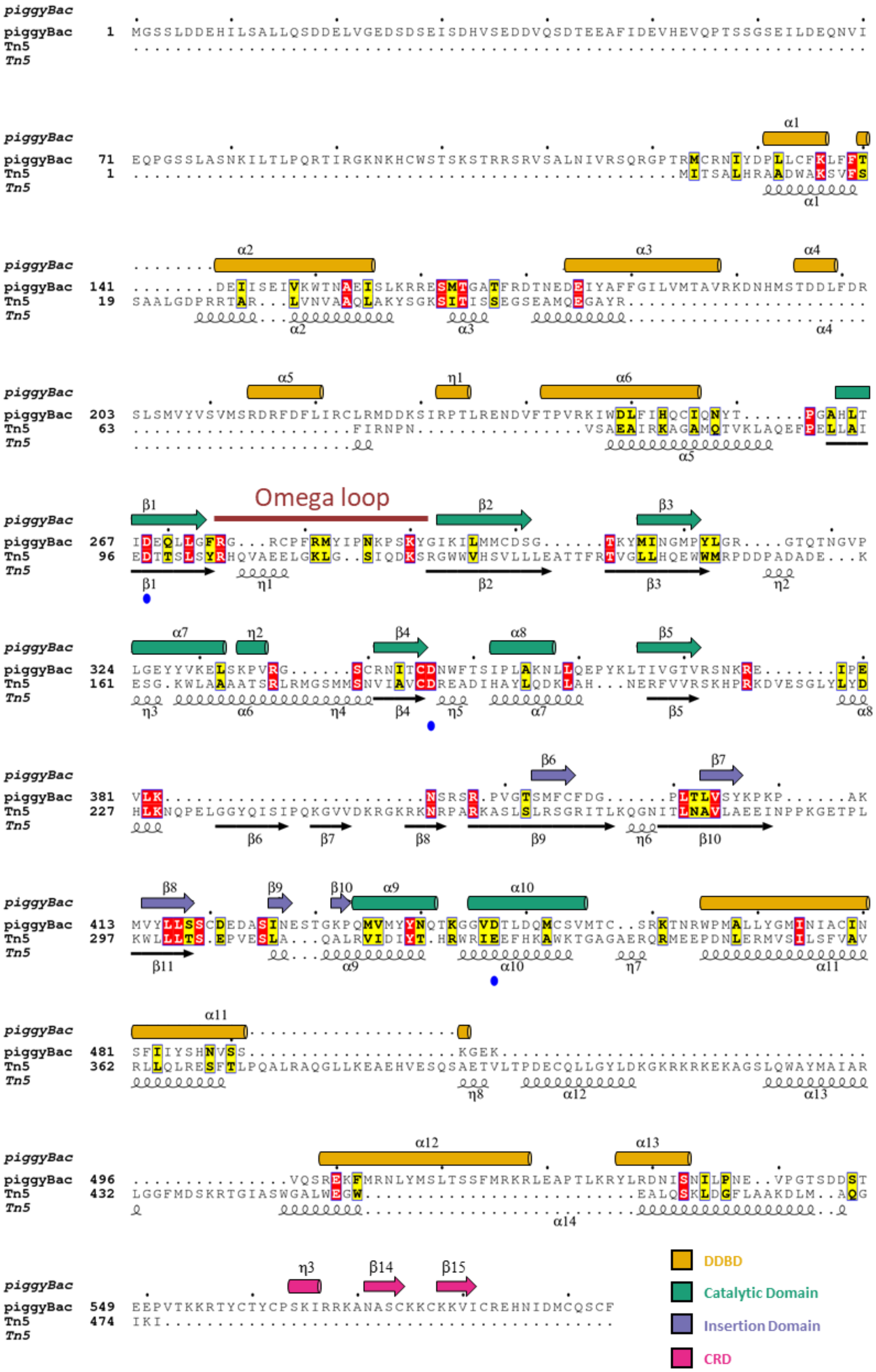


**Supplementary Figure 7. Structure-based sequence alignment of PB and Tn*5* transposases**

The alignment was performed using the pairwise structure comparison module of the DALI server (http://ekhidna2.biocenter.helsinki.fi/dali/). The DALI result was used to generate the

alignment using ESPript 3.0 (http://espript.ibcp.fr/). The secondary structural elements of PB are highlighted with the color scheme defined in Fig. 1c. The CRD secondary structural elements are as determined from the NMR model (PDB: 5LME).

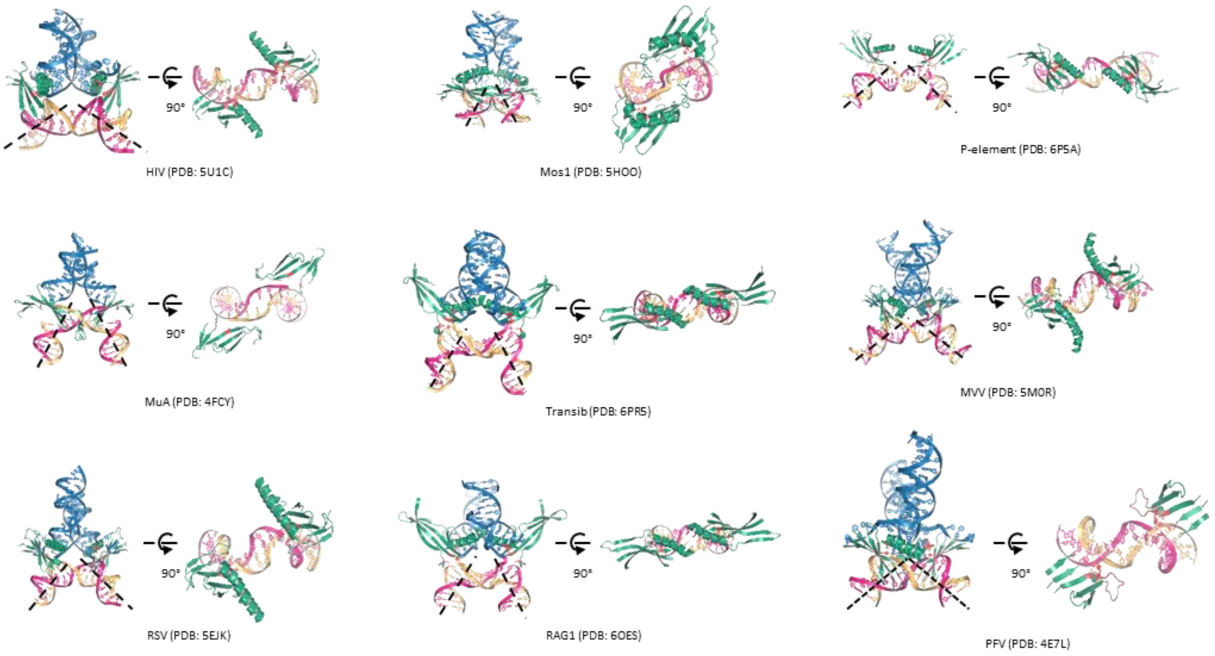


**Supplementary Figure 8. STC structures of intasomes and transpososomes.**

HIV: human immunodeficiency virus. MVV: maedi-visna virus. RSV: Rous sarcoma virus. RAG: Recombination-activating gene. PFV: prototype foamy virus.

**Supplementary Table 1. Cryo-EM data collection, refinement and validation statistics**

| | SNHP (EMD-22073) (PDB 6X68) | STC (EMD-22072) (PDB 6X67) |
|---|---|---|
| **Data collection and processing** | | |
| Magnification | 130,000 | 130,000 |
| Voltage (kV) | 300 | 300 |
| Electron exposure ($e^{-}/Å^2$) | 73.7 | 46.6 |
| Defocus range (μm) | -1.0 -- -2.0 | -1.0 -- -2.0 |
| Pixel size (Å) | 1.06 | 1.06 |
| Symmetry imposed | C1 | C1 |
| Initial particle images (no.) | 2,623,168 | 6,723,784 |
| Final particle images (no.) | 35,960 | 43,512 |
| Map resolution (Å) | 3.66 | 3.47 |
| FSC threshold | 0.143 | 0.143 |
| Map resolution range (Å) | 3.50 – 5.87 | 3.27 – 5.65 |
| | | |
| **Refinement** | | |
| Initial model used (PDB code) | 5LME and *ab initio* | SNHP |
| Model resolution (Å) | 3.7 | 3.5 |
| FSC threshold | 0.5 | 0.5 |
| Model resolution range (Å) | 3.50 – 5.87 | 3.27 – 5.65 |
| Map sharpening *B* factor ($Å^2$) | -109 | -91 |
| Model composition | | |
| Non-hydrogen atoms | 10244 | 10982 |
| Protein residues | 956 | 956 |
| Nucleotides | 124 | 160 |
| Ligands | Zn: 4, Ca: 4 | Zn: 4, Ca: 4 |
| *B* factors ($Å^2$) | | |
| Protein | 65.03 | 81.87 |

| | | |
|---|---|---|
| Nucleic acid | 99.19 | 154.14 |
| Ligand | 144.48 | 126.65 |
| R.m.s. deviations | | |
| Bond lengths (Å) | 0.017 | 0.024 |
| Bond angles (°) | 1.758 | 1.806 |
| Validation | | |
| MolProbity score | 1.08 | 1.58 |
| Clashscore | 2.32 | 4.62 |
| Poor rotamers (%) | 0.11 | 0.00 |
| Ramachandran plot | | |
| Favored (%) | 97.69 | 95.06 |
| Allowed (%) | 2.0 | 4.31 |
| Disallowed (%) | 0.32 | 0.63 |
