# Supplementary material for: Structural basis of seamless excision and specific targeting by piggyBac transposase
Source: Nat Commun. 2020 Jul 10;11:3446. doi: 10.1038/s41467-020-17128-1 (PMC7351741; doi:10.1038/s41467-020-17128-1)
Supplement: Supplementary file 2 — Peer Review [file 41467_2020_17128_MOESM2_ESM.pdf]

REVIEWERS' COMMENTS:

Reviewer #1 (Remarks to the Author):

This manuscript presents the cryoEM structures of piggyBAC transpososomes in two different reaction states although with supporting biochemistry.
The structures are very intriguing and add new features and insights to the transpososome zoo. As the authors point out, one aspect of piggyBAC that is useful in biotech is that it leaves its previous host-DNA location with 4bp sticky ends that can be simply ligated together, leaving no trace that the transposon was ever there at all, and without requiring error-prone repair processes. This is quite unusual among transposases and the structure reveals how it does this. Additionally, the authors were able to design new transposon ends that not only support their hypotheses but also enhance activity.

Although the biochemistry appears to be fine and is not the primary "new knowledge" of the paper, it would be helpful if it were described and discussed more clearly.

Specific points, mostly aimed at clarity not substance:

Is the density clear enough to support all the H-bonds shown in Figures 4d and e and 5?

For all the biochemistry figures (including the supplementary ones), the legends are excessively concise. Please add concentrations of proteins and DNAs, and a bit more information in general.

The structural work is described in refreshingly useful detail.

Supplementary figure 1b: Please label the lanes and label which cartoon pertains to which lane. It took some time to figure out that the cartoon between gels refers to the right lane of each gel rather than a different experiment run on the right gel.

Figure s1c and d: it would be nice to see all the variations on the ends tested side-by-side in the same assay rather than the different assays used in parts c and d.
I can't find the methods for the experiment in Figure s1d, and the diagram is puzzling: if pUC was linearized by XmaI digest, what are the nicks at each end? If this is an ethidium-stained gel then why do some DNA ends have little stars on them?
The only activity I can see is in lane 3, so why does the legend specify that little activity was seen in lane 2 when there's no decrease in substrate in lane 1 either?
What is the schmutz in the bottom of lane 1?
If only 4 sites in pUC19 are used as targets as the 2nd paragraph of the methods states, why so many bands in lane 3?

The product-sequencing is a minimally-consequential part of this paper - the authors should be praised for completeness in doing it even though it is simply confirms they see with their transposon ends what other groups have seen before for piggyBAC. That said, it could be better-described and better documented (it is essentially all "data not shown").
The 1st paragraph of "results" implies that what was sequenced was the products of LE35 oligonucleotide insertion, but the 2nd paragraph of the methods section says that for sequencing a completely different in vivo transposition reaction was done using LE35 plus RE63 on the two ends of the (sub-minimally described) mini-transposon. (that paragraph could also use a topic sentence: one doesn't know what experiment is being described until the end).

P7, bottom – It makes sense that the target-binding channel narrows between the hairpin and STC structures, but could it also be that the channel is simply flexible, and other classes of particles that were discarded during processing had other widths?

P8, middle – worth noting that the there is an A-tract between the two CRD-binding sites, which would naturally tend to narrow the minor groove (if I remember correctly, 5’A-3’T steps don’t disrupt A-tract structures). In Figure 3b it seems to adopt a cannonical A-tract structure with propeller twist as well as narrow minor groove – is that true?

Figure 4d vs. the text describing it on the top of p10 puzzle me sorely. I can’t find Y439 in the figure, although Y406 is shown (blue over blue, hard to see). In the view shown, Y283 doesn’t seem to be near anything interesting. I also don’t see the other residues involved in the methyl-binding pocket. It might be worth making a closer-up version of d and e? It might lose context but the important details might show better.

In figure 4 the number schemes for target vs. hairpin nucleotides are confusing, both schemes conflict with figure 4g, and neither are explained until figure 5.

Figure 4f is really cool and worth a little more weight in the text!

The mode of target recognition is very interesting and surprising.

P12: please give a bit more explanation of the “colony count” assay – even if it is previously published, one shouldn’t have to read the reference list to figure out the basics of what’s going on here. Both the figure legends and methods are rather cryptic. What PCR primers were used? What is pTpB? What is the significance of resistance to G418? Etc. Also, the figure would be a little more at-a-glance comprehensible if the 2nd panels were labeled “insertion” rather than “colony count”. Or is the “colony count” assay looking at complete transposition from one location to another? Why do the excision vs. colony count assays give somewhat different answers? Or are both panels measuring excision only (from a G418-resistance gene)?

P12, last paragraph: I don’t understand the argument about how the fact RE 1-33 is functional in vitro supports the formation of tetramers in vivo. Yes, the part colored black could in a pinch still act as a binding site for the CRD – but wouldn’t that just make a dimeric complex exactly as shown in your nice EM structures? The first sentence on p12 implies that simply adding more protein might enhance activity (although the phrase “away from the transposon tip and its site of catalysis” is awkwardly placed) – was that tried?

Is there any evidence for contacts between the CRDs and the rest of the transposase as hinted at in figure 6c? Such contacts wouldn’t be necessary for the model, but without them I wouldn’t call it a “tetramer” – just two dimers.

Figure 6e is very clever and potentially quite useful.

Typos, etc:
Abstract, near end: “synaptic formation” should be synaptic complex formation”
Figures 2c and b are swapped.
P11, 1st paragraph: “with which R281 and Y291 interact …” is a misplaced modifier.
P12, 1st full paragraph – “dimeric” should be “dimer”
P14, bottom: “recognition and integration is” should be “… are”, and I think “in vitro” should be in front of “reactions involving hairpin formation …”

Reviewer #2 (Remarks to the Author):

The PiggyBac (PB) transposon is a prominent genome engineering tool used in transgenesis, genetic

screening, stem cell biology and gene therapy. Given its applied significance, a mechanistic understanding of the PB transposition pathway has been long sought. In this manuscript, Chen et al. present the first structural views of PB. Using cryoEM, the authors describe two structures of the PB transposase: one in complex with excised transposon end DNA (SNHP) and one with the transposon ends inserted into the cognate genomic target sequence (STC). Unexpectedly, the structures reveal an asymmetric molecular arrangement, which helps explain PBs unique features. Using cleverly designed in vitro and in vivo transposition assays, the authors support the notion that structural asymmetry promotes transposon end recognition and synapsis, supporting PBs activity in cells. Furthermore, the work elucidates the structural principles of PB's target site selection and sheds new light onto transposase hyperactivity.
Overall, this study presents important insights into a process of high biotechnological and medical relevance. The experiments were carefully designed and conducted, and the paper is very well written and easy to follow. Undoubtedly, the presented results will be of broad interest; they will likely provide inspiration for studying diverse transposons and for improving their use in genome engineering. Thus, I only have a few minor questions and suggestions, which the authors may wish to consider.

- In the abstract: "The results show that the structure of the excision intermediate and the precision of TTAA targeting create the link that give rise to the specific properties of piggyBac." It is not easy to appreciate the essence of this sentence for the uninformed reader, please revise.
- Second paragraph of the introduction: The term "genome editing" is predominantly used when changes are made on a site-directed manner (e.g. in the context of CRISPR/Cas-, ZFN- or TALEN-mediated modifications). As SB- and PB-mediated modifications occur throughout the genome, alternative terms - such as genome engineering or gene insertion - seem more appropriate.
- Did the SNHP structure provide any insights into cleavage site selection on the NTS? Why does the 3'OH of the TS attack the NTS exactly 4nts into the flanking DNA, and how does it come there? Can the mechanism be similar to what was seem for Hermes and RAG? Also, does the structure reveal why the size of the hairpin tip differs in Tn5 and PB?
- Page 10, last paragraph: As the two strands of the TTAA target sequence are located on separate DNA molecules, it is not immediately obvious why the authors have expected them to pair. Perhaps it will be worthwhile to refer to STC complex structures of Mu and Mos1, where the same DNA design yielded base paired target sites to clarify this point.
- On a related point, the authors suggest that TTAA is already melted in the target DNA prior to strand transfer. Do the structures provide any clues for what can drive such distortions in dsDNA?
- Page 11, 2nd paragraph: "This unusual mode of transposon target selection is in line with our inability to identify any part of PB that would recognize the TTAA target in dsDNA form." Please clarify if this comment refers to structure analysis or experimental data.
- Regarding the in vivo transposition assays, can the authors clarify why additional CRD binding sites in LE increase PB activity?
- On page 14, the discussion regarding structural coupling of the transposition steps in PB and other systems is interesting, but could perhaps be extended a bit. How are the steps of PB more linked than of other elements and how exactly is this connected to seamless excision? Perhaps contrasting to a different element could be helpful. If I understand correctly, PB's main trick is to excise through a 4 nt hairpin intermediate, exactly embracing its palindromic TSD, which leaves complementary overhangs on the flanks for seamless repair.
- Can the authors speculate why hyPBase is less dependent on transposon end asymmetry?

REVIEWER #1
This manuscript presents the cryoEM structures of piggyBAC transpososomes in two different reaction states although with supporting biochemistry.
The structures are very intriguing and add new features and insights to the transpososome zoo. As the authors point out, one aspect of piggyBAC that is useful in biotech is that it leaves its previous host-DNA location with 4bp sticky ends that can be simply ligated together, leaving no trace that the transposon was ever there at all, and without requiring error-prone repair processes. This is quite unusual among transposases and the structure reveals how it does this. Additionally, the authors were able to design new transposon ends that not only support their hypotheses but also enhance activity.

Although the biochemistry appears to be fine and is not the primary "new knowledge" of the paper, it would be helpful if it were described and discussed more clearly.
As suggested by the reviewer, we have rewritten the section related to the biochemical results shown in Supplementary Figure 1 and have added a brief paragraph describing the mini-transposon integration assay and results. These is accompanied by a more detailed legend.

Specific points, mostly aimed at clarity not substance:
Is the density clear enough to support all the H-bonds shown in Figures 4d and e and 5?
The regions displayed in old Figures 4d and e (now Figures 4b and c) correspond to the highest quality potential density of the structures. In these regions, the indicated H-bond lengths differ only about 0.2Å between the two monomers (they should be very similar, of course) implying the approximate measure coordinate precision. As for the schematics in Figure 5, we are confident in the interactions as indicated. Certainly because of the relatively poorer quality of the potential density in the CRDs, both the coordinate accuracy and precision are worse; nevertheless, we are confident that the indicated interactions exist.

For all the biochemistry figures (including the supplementary ones), the legends are excessively concise. Please add concentrations of proteins and DNAs, and a bit more information in general.
Within the constraints of ~350 words, we have revised the figure legends of Figures 6a, b and d with more details about the excision and colony count transposition assays, as well as data analysis methods. As indicated above, we have also substantially revised the figure legend of the main biochemical figure, Supplementary Figure 1, to include the information requested as well as a brief description of each biochemical assay.

The structural work is described in refreshingly useful detail.
Thank you!

Supplementary figure 1b: Please label the lanes and label which cartoon pertains to which lane. It took some time to figure out that the cartoon between gels refers to the right lane of each gel rather than a different experiment run on the right gel.
We apologize for the confusing data presentation. The layout of the panel has been modified such that the lane numbers on the top of both gels correspond to the designated reaction diagrams on the left. Diagram 1 illustrates the second strand cleavage activity and diagram 2 illustrates hairpin opening activity. The two gel panels have been moved together and the DNA marker indicators are arranged on the left hand side of the gel. This information has also been added to the revised figure legend.

Figure s1c and d: it would be nice to see all the variations on the ends tested side-by-side in the same assay rather than the different assays used in parts c and d.
We agree with the reviewer that the variations on the RE TIR ends (old Supplementary Figure 1d) should be tested in the same assay as that in Supplementary Figure 1c in which supercoiled pUC19 was used as target DNA substrate. We have therefore replaced the panel in Supplementary Figure 1d with one in

which we tested a wider set of RE TIR end variations on both linearized and supercoiled pUC19. The main result - that shortening RE63 leads to higher activity - remains unchanged.

I can't find the methods for the experiment in Figure s1d, and the diagram is puzzling: if pUC was linearized by XmaI digest, what are the nicks at each end? If this is an ethidium-stained gel then why do some DNA ends have little stars on them?
We thank the referee for noting the missing methods corresponding to Supplementary Figure 1d; they have now been added to the methods section under the heading "In vitro transposition assay using TIR DNA".

As for the diagram in the original text, we inadvertently used an old diagram which depicted linearized pUC19 being generated by integrating fluorescently labelled TIR DNA (indicated by the stars) into supercoiled pUC19 (i.e., equivalent to the DE product of supplementary figure 1c) - and hence the nicks. We are sorry for the confusion and have corrected the diagram to reflect that the correct target substrate is pUC19 linearized by XmaI digestion.

The only activity I can see is in lane 3, so why does the legend specify that little activity was seen in lane 2 when there's no decrease in substrate in lane 1 either?
We thank the reviewer for pointing this out. The panel in question has now been replaced in response to the reviewer's earlier comment. The legend has also been modified.

What is the schmutz in the bottom of lane 1?
The schmutz was a result of cropping the bottom of the original gel to remove the signal due to unintegrated oligos and completely fragmented pUC19, and that in lane 1 corresponded to RE TIR 1-63 DNA, the longest TIR among these three tested variations. We now explicitly address the schmutz in the revised figure legend to Supplementary Figure 1d: "The smeared DNA on the bottom is a mixture of small truncated DNA resulting from pUC19 cleavage and the free RE TIR substrate."

If only 4 sites in pUC19 are used as targets as the 2nd paragraph of the methods states, why so many bands in lane 3?
We apologize for the confusion. We should have been clearer that we only detected integration into 4 of the possible 13 sites. Had we sequenced more colonies, we probably would have detected integration into others as well (excluding those in the Amp or *ori* genes). In the experiment that was shown in Supplementary Figure 1d, we used a solo TIR DNA for integration instead of a mini-transposon in which two TIRs are coupled and flank a resistance gene. Integration of solo TIR DNA will generate many DSBs, and given that there are 13 TTAA in pUC19 as potential DSB sites and combinations of them, we see many DSB bands under this experimental setup.

The product-sequencing is a minimally-consequential part of this paper - the authors should be praised for completeness in doing it even though it is simply confirms they see with their transposon ends what other groups have seen before for piggyBAC. That said, it could be better-described and better documented (it is essentially all "data not shown").
We thank the reviewer for the kind words. We have added a short paragraph to the first section of the results which describes the assay and the specific targeting results as follows: "To confirm that mammalian-expressed PB integrated with its expected target site specificity, we generated a linear mini-transposon in which LE35 and RE63 TIRs flanked a Kan resistance gene, and used this as a substrate for *in vitro* integration into SC pUC19. The reaction products were purified and transformed into *E. coli*, allowing us to select for Amp+Kan+ colonies corresponding to integrated mini-transposons. Sequencing confirmed that only TTAA tetranucleotides had been targeted: from ten sequenced colonies, we detected four mini-transposon integration events corresponding to insertion at the TTAA sequences at bp 635-638, bp 1568-1571, bp 1582-1585, and bp 2646-2649 of pUC19."

The 1st paragraph of "results" implies that what was sequenced was the products of LE35 oligonucleotide insertion, but the 2nd paragraph of the methods section says that for sequencing a completely different in vivo transposition reaction was done using LE35 plus RE63 on the two ends of the (sub-minimally described) mini-transposon. (that paragraph could also use a topic sentence: one doesn't know what experiment is being described until the end).
We apologize for the poorly presented section. As indicated above, we have added a short paragraph to the first section of the result to clearly state what was sequenced. We have also added a subtitle in the methods section: "In vitro transposition assays using a mini-transposon".

P7, bottom - It makes sense that the target-binding channel narrows between the hairpin and STC structures, but could it also be that the channel is simply flexible, and other classes of particles that were discarded during processing had other widths?
We agree with the reviewer that we cannot rule out the possibility that classes with different channel widths exist in the data set. We have revised the text by adding: "...., although we cannot rule out the possibility that this observation was influenced by the selection of the 3D classes."

P8, middle - worth noting that the there is an A-tract between the two CRD-binding sites, which would naturally tend to narrow the minor groove (if I remember correctly, 5'A-3'T steps don't disrupt A-tract structures). In Figure 3b it seems to adopt a cannonical A-tract structure with propeller twist as well as narrow minor groove - is that true?
We thank the reviewer for pointing this out. Indeed, DNA bps 22-25 contains a canonical A-tract that adopts a propeller twist and a narrowed minor groove. We now note this in the text and have added a reference to Stefl et al., PNAS (2004) which describes features of A-tract DNA.

Figure 4d vs. the text describing it on the top of p10 puzzle me sorely. I can't find Y439 in the figure , although Y406 is shown (blue over blue, hard to see). In the view shown, Y283 doesn't seem to be near anything interesting. I also don't see the other residues involved in the methyl-binding pocket . It might be worth making a closer-up version of d and e? It might lose context but the important details might show better.
We agree with the reviewer that these are important interactions and we apologize for the less-than-optimal original presentation. We agree with the reviewer that zoomed-in views are better, and therefore we have added the appropriate residues to larger versions of Figure 4d and e, and accompany them with close-up views of the methyl-binding pocket. To make space, we have moved old Figures 4a,b,c to new Supplementary Figure 6; old Figure 4d and e are now Figure 4b and c. We have also moved the label "Y406" to avoid any overlap.

In figure 4 the number schemes for target vs. hairpin nucleotides are confusing, both schemes conflict with figure 4g, and neither are explained until figure 5.
The reviewer is correct and we should have introduced the number schemes for target and hairpin nucleotides before showing the structure. We have added a schematic explaining the number schemes to new Figure 4a.

Figure 4f is really cool and worth a little more weight in the text!
Thank you, we think it's really cool as well! With the reviewer's encouragement, we have added these sentences to the text: "To allow the reaction to proceed from the hairpin-bound state to one poised to capture target, it appears that hairpin resolution is followed by the movement of the resulting flap out of the active site. Then, a drastically distorted and unpaired target TTAA tetranucleotide can be bound. As the structures reveal, the key to *pB* transposition is that the backbones of the TTAA tetranucleotide in both the hairpin and target DNA adopt a very similar conformation (Fig. 4d). The role of the PB protein is therefore to enforce this conformation at both steps of the reaction."

The mode of target recognition is very interesting and surprising.

P12: please give a bit more explanation of the "colony count" assay - even if it is previously published, one shouldn't have to read the reference list to figure out the basics of what's going on here. Both the figure legends and methods are rather cryptic.
We agree with the reviewer that we had been too brief in our introduction and discussion of the colony count assay, and we have modified the first paragraph of the "pB transposition in cells" section as follows:
"We used colony count and excision assays in cultured human cells using PB and *pB* transposon derivatives as a proxy for in vivo transposition[64]. Excision analysis uses PCR to amplify re-joined transposon plasmid ends recovered from transfected cells indicating transposon excision has occurred. The colony count assay involves excision of a neomycin resistance transposon (pTpB) from a transposon plasmid followed by integration into the genomes of cells. Cells that have undergone transposition grow and form colonies in the presence of G418 which allows selection for the neomycin resistance gene, thereby providing a quantitative readout of not only excision but also subsequent integration."

New details have also been added to the methods as follows in bold:
"pCMV-HA-piggyBac (PB)[89] **encodes a hemagglutin (HA)-tagged PB transposase**. All transposon plasmids were derived from pTpB[64] using standard molecular biology techniques. All plasmids were confirmed by DNA sequencing. For excision assays, 3 million HT-1080 cells were seeded into a 100mm dish. The next day, cells were transfected with 10 µg of the transposon and 5 µg transposase plasmids using lipofectamine (ThermoFisher). One day later, cells were trypsinized and excision analysis **PCR** was performed **using the following primers (forward: 5'-ATGCGGCATCAGAGCAGATT-3', reverse: 5'-TGTGTGGAATTGTGAGCGGA-3')**[64]. For colony counts, 0.4 million HT-1080 cells were seeded into each well of a 6-well plate. The next day, cells were transfected with 1 µg of transposon and 0.5 µg of transposase plasmids using lipofectamine. One day later, cells were trypsinized and diluted to 100mm dishes followed by selection with 600 µg/ml of the **antibiotic** G418 for 8-10 days. **Colonies of cells were fixed in 10% formaldehyde/phosphate-buffered saline (PBS), stained with 1% methylene blue in PBS, and counted**[90]**.**"

What PCR primers were used? These are now specified in the revised methods section.
What is pTpB? This is now defined in the new results section.
What is the significance of resistance to G418? This is now specified above.
Also, the figure would be a little more at-a-glance comprehensible if the 2nd panels were labeled "insertion" rather than "colony count". We agree with the reviewer. The requested change has been made.
Or is the "colony count" assay looking at complete transposition from one location to another? Why do the excision vs. colony count assays give somewhat different answers? Or are both panels measuring excision only (from a G418-resistance gene)? We trust some of these questions have been answered in the new section. Different answers arise because the colony count is measuring "not only excision but also subsequent integration."

P12, last paragraph: I don't understand the argument about how the fact RE 1-33 is functional in vitro supports the formation of tetramers in vivo. Yes, the part colored black could in a pinch still act as a binding site for the CRD - but wouldn't that just make a dimeric complex exactly as shown in your nice EM structures? The first sentence on p12 implies that simply adding more protein might enhance activity (although the phrase "away from the transposon tip and its site of catalysis" is awkwardly placed) - was that tried?
We apologize for not being clearer in the text. The reviewer is absolutely right that we believe RE 1-33 makes a dimeric complex exactly as seen in the structures. The point we should have emphasized was that it is the relative activity of RE 1-33 vs. RE 1-63 that implicates two dimers. In such a model, one dimer binds the inner site of the RE due to high binding affinity of the CRD to RE 45-63, leaving space for

another dimer to occupy RE1-44. The reviewer is correct that further experimental work both *in vitro* and *in vivo* is required to investigate the validity of the model; however, we believe that these would be beyond the scope of the current paper. We have revised the end of the paragraph as follows (and have deleted the awkward phrase altogether):
"This cryptic CRD binding site may be sufficient to allow the assembly of a RE33/RE33 complex analogous to that on LE35/LE35, a combination that is also active *in vitro*. **The most reasonable explanation** for the inhibition **observed** when RE33 is extended to RE63 is **that** bp 34-63 of the RE are almost identical to LE bp 7-35 (Fig. 1b); **thus,** under our assay conditions **with limiting protein**, they provide a competing binding site for a PB dimer. **This is consistent with a second dimer binding site in the authentic LE/RE PB transpososome.**"

Is there any evidence for contacts between the CRDs and the rest of the transposase as hinted at in figure 6c? Such contacts wouldn't be necessary for the model, but without them I wouldn't call it a "tetramer" - just two dimers.
We agree with the reviewer that we do not have any strong evidence to distinguish a tetramer from two dimers that do not contact each other. It seemed wisest therefore to delete the "tetramer" terminology throughout.

Figure 6e is very clever and potentially quite useful.
We hope so.

Typos, etc:
Abstract, near end: "synaptic formation" should be synaptic complex formation"
It has been corrected.
Figures 2c and b are swapped.
We thank the reviewer for catching this. We have fixed them in the text to reflect the correct order.
P11, 1st paragraph: "with which R281 and Y291 interact ..." is a misplaced modifier.
We have removed reference to these residues in the text.
P12, 1st full paragraph - "dimeric" should be "dimer"
This has been corrected.
P14, bottom: "recognition and integration is" should be "... are", and I think "in vitro" should be in front of "reactions involving hairpin formation ..."
We agree. The text has been corrected accordingly.

####################
REVIEWER #2 (Remarks to the Author):

The PiggyBac (PB) transposon is a prominent genome engineering tool used in transgenesis, genetic screening, stem cell biology and gene therapy. Given its applied significance, a mechanistic understanding of the PB transposition pathway has been long sought. In this manuscript, Chen et al. present the first structural views of PB. Using cryoEM, the authors describe two structures of the PB transposase: one in complex with excised transposon end DNA (SNHP) and one with the transposon ends inserted into the cognate genomic target sequence (STC). Unexpectedly, the structures reveal an asymmetric molecular arrangement, which helps explain PBs unique features. Using cleverly designed in vitro and in vivo transposition assays, the authors support the notion that structural asymmetry promotes transposon end recognition and synapsis, supporting PBs activity in cells. Furthermore, the work elucidates the structural principles of PB's target site selection and sheds new light onto transposase hyperactivity.

Overall, this study presents important insights into a process of high biotechnological and medical relevance. The experiments were carefully designed and conducted, and the paper is very well written and easy to follow. Undoubtedly, the presented results will be of broad interest; they will likely provide

inspiration for studying diverse transposons and for improving their use in genome engineering. Thus, I only have a few minor questions and suggestions, which the authors may wish to consider.

- In the abstract: "The results show that the structure of the excision intermediate and the precision of TTAA targeting create the link that give rise to the specific properties of piggyBac." It is not easy to appreciate the essence of this sentence for the uninformed reader, please revise.
As suggested, we have revised the sentence. It now reads: "The results show that the excised TTAA hairpin intermediate and the TTAA target adopt essentially identical conformations, providing a mechanistic link connecting the two unique properties of *piggyBac*."

- Second paragraph of the introduction: The term "genome editing" is predominantly used when changes are made on a site-directed manner (e.g. in the context of CRISPR/Cas-, ZFN- or TALEN-mediated modifications). As SB- and PB-mediated modifications occur throughout the genome, alternative terms - such as genome engineering or gene insertion - seem more appropriate.
We agree with the reviewer that "genome engineering" is better in this context, and we have revised the sentence accordingly.

- Did the SNHP structure provide any insights into cleavage site selection on the NTS ? Why does the 3'OH of the TS attack the NTS exactly 4nts into the flanking DNA, and how does it come there? Can the mechanism be similar to what was seem for Hermes and RAG? Also, does the structure reveal why the size of the hairpin tip differs in Tn5 and PB?
The reviewer brings up interesting and insightful questions. The selection of the scissile phosphate exactly 4nts into flank is governed by the stabilization of exactly 4 unpaired bases in the hairpin loop by the transposase.

Considering that the TS is very unlikely to move out of the active site, indeed the most parsimonious notion is that the NTS must undergo a major conformational change to bring the scissile phosphate to the active site, perhaps along the lines that seen in the RAG1 and Hermes cases but with the opposed polarity. However, as at this point we do not have experimental information regarding the steps prior to hairpin formation, we would prefer not to speculate on this issue.

There are two main differences between the Tn*5* and PB arrangements. One is that the PB active site area has more room to accommodate the longer hairpin than that of Tn*5*, and the second is that the set of omega loop interactions *in trans* that stabilize the exactly 4nt long loop in PB are completely absent in Tn*5*. In fact, these two observations are also consistent with the idea that a relatively large motion of the NTS has to occur to bring the scissile phosphate to the PB active site. We have added two additional sentences in Results at the end of "SNHP complex and DNA hairpin recognition" section to explain these points better. The sentences are as follows: "The active site area of PB is more open when compared to that of Tn*5*, consistent with the ability to accommodate a longer hairpin loop and to allow the conformation change of the NTS that might be required to bring the scissile phosphate to the active site. The 4 nt long hairpin loop is stabilized by a set of interactions with the omega loop *in trans*, which are absent in Tn*5*.

- Page 10, last paragraph: As the two strands of the TTAA target sequence are located on separate DNA molecules, it is not immediately obvious why the authors have expected them to pair. Perhaps it will be worthwhile to refer to STC complex structures of Mu and Mos1, where the same DNA design yielded base paired target sites to clarify this point.
Our expectation was precisely driven by other transpososome structures such as that of Mu and several intasomes as well, where the target sites while distorted are base paired. (In the Mos1 STC complex, the TA target site is not base paired.) Now we know from the pB STC complex that the target-site specificity that sets pB apart from the others is mediated by the recognition of the unpaired ssDNA form of the

TTAA target and this why the 4 bps are unpaired. We believe that the design of the STC substrate should not affect the architecture of the STC.

- On a related point, the authors suggest that TTAA is already melted in the target DNA prior to strand transfer. Do the structures provide any clues for what can drive such distortions in dsDNA?
Yes! The structures show that the active sites are located very deep inside the catalytic domains, necessitating a sharp bend in target DNA. In addition, it appears that the omega loops of PB play a key role in inserting into the minor groove of the target DNA to facilitate unpairing. We have added the following to page 12. “Altogether, the unique aspects of PB target DNA recognition and integration are driven by the location of the active sites within the dimer, the interactions of the omega loops, and the relative positions of the target scissile bonds."

- Page 11, 2nd paragraph: "This unusual mode of transposon target selection is in line with our inability to identify any part of PB that would recognize the TTAA target in dsDNA form." Please clarify if this comment refers to structure analysis or experimental data.
We apologize for being imprecise. Our conclusion is based on the analysis of the STC structure and was not intended as a comment on experimental limiations. What we intended to convey was that our structural data did not indicate the presence of a protein domain or subdomain of PB that might be responsible for specific target recognition. We have rephrased the sentence as follows: "This unusual mode of transposon target selection is in line with the lack of a region of PB recognizable in the structure as a potential dsDNA TTAA target recognition domain."

- Regarding the in vivo transposition assays, can the authors clarify why additional CRD binding sites in LE increase PB activity?
We suspected that additional CRD binding sites would promote synaptic complex formation in cells, thus increasing the PB activity. We have added the following to the end of the in vivo transposition section: "..., suggesting that an additional dimer binding site stimulates activity, perhaps by promoting the formation of the synaptic complex."

- On page 14, the discussion regarding structural coupling of the transposition steps in PB and other systems is interesting, but could perhaps be extended a bit. How are the steps of PB more linked than of other elements and how exactly is this connected to seamless excision? Perhaps contrasting to a different element could be helpful. If I understand correctly, PB's main trick is to excise through a 4 nt hairpin intermediate, exactly embracing its palindromic TSD, which leaves complementary overhangs on the flanks for seamless repair.
The reviewer is absolutely correct, and we now contrast the transposition steps of PB to another element. We have expanded the discussion along the lines of the reviewer's suggestion: “For example, the *SB* transposon is excised with 3’ overhangs because cleavage on the NTS is 3-nt within the transposon itself. The resulting 3-nt CAG overhangs at the donor site are not complementary and when the DSB is repaired, an excision footprint is left that also includes one extra copy of the TA target site of SB.[9]”

- Can the authors speculate why hyPBase is less dependent on transposon end asymmetry?
The reviewer brings up an interesting question and we wish we had a structural answer. One possibility is that the seven point mutations in hyPBase allows it to form a more stable protein-DNA complex, relaxing the stringent site structure requirements of WT PB. Our current plans include structural work on hyPBase and experiments are currently underway.
